# Randomized controlled pilot of a group antenatal care model and the sociodemographic factors associated with pregnancy-related empowerment in sub-Saharan Africa

**DOI:** 10.1186/s12884-017-1493-3

**Published:** 2017-11-08

**Authors:** Crystal L. Patil, Carrie S. Klima, Sebalda C. Leshabari, Alana D. Steffen, Heather Pauls, Molly McGown, Kathleen F. Norr

**Affiliations:** 1 Department of Women, Children and Family Health Science, College of Nursing, University of Illinois at Chicago, Chicago, IL USA; 2 School of Nursing, Muhimbili University of Health and Allied Sciences, Dar es Salaam, Tanzania; 3 Department of Health Systems Science, University of Illinois at Chicago, Chicago, IL USA; 4 Office of Research Facilitation, University of Illinois at Chicago, Chicago, IL USA; 5 Institute for Public Health and Medicine, Northwestern University, Chicago, IL USA

## Abstract

**Background:**

The links between empowerment and a number of health-related outcomes in sub-Saharan Africa have been documented, but empowerment related to pregnancy is under-investigated. Antenatal care (ANC) is the entry point into the healthcare system for most women, so it is important to understand how ANC affects aspects of women’s sense of control over their pregnancy. We compare pregnancy-related empowerment for women randomly assigned to the standard of care versus CenteringPregnancy-based group ANC (intervention) in two sub-Saharan countries, Malawi and Tanzania.

**Methods:**

Pregnant women in Malawi (*n* = 112) and Tanzania (*n* = 110) were recruited into a pilot study and randomized to individual ANC or group ANC. Retention at late pregnancy was 81% in Malawi and 95% in Tanzania. In both countries, individual ANC, termed focused antenatal care (FANC), is the standard of care. FANC recommends four ANC visits plus a 6-week post-birth visit and is implemented following the country's standard of care. In group ANC, each contact included self- and midwife-assessments in group space and 90 minutes of interactive health promotion. The number of contacts was the same for both study conditions. We measured pregnancy-related empowerment in late pregnancy using the Pregnancy-Related Empowerment Scale (PRES). Independent samples *t*-tests and multiple linear regressions were employed to assess whether group ANC led to higher PRES scores than individual ANC and to investigate other sociodemographic factors related to pregnancy-related empowerment.

**Results:**

In Malawi, women in group ANC had higher PRES scores than those in individual ANC. Type of care was a significant predictor of PRES and explained 67% of the variation. This was not so in Tanzania; PRES scores were similar for both types of care. Predictive models including sociodemographic variables showed religion as a potential moderator of treatment effect in Tanzania. Muslim women in group ANC had a higher mean PRES score than those in individual ANC; a difference not observed among Christian women.

**Conclusions:**

Group ANC empowers pregnant women in some contexts. More research is needed to identify the ways that models of ANC can affect pregnancy-related empowerment in addition to perinatal outcomes globally.

**Electronic supplementary material:**

The online version of this article (doi:10.1186/s12884-017-1493-3) contains supplementary material, which is available to authorized users.

## Background

Empowerment is a complex multidimensional concept that can be broadly defined as the ability of individuals or groups “*to improve capacities, to critically analyze situations and to take actions to improve those situations*” [1]. In sub-Saharan Africa, women’s overall empowerment has been positively associated with the utilization of maternal health services [2], use of contraception [3–5], improved infant feeding practices [6], and reductions in infant mortality [7].

Given the positive associations between general empowerment and maternal-child health, it is important to consider women’s empowerment as it relates to health and healthcare [8]. Health-related empowerment is a construct that was developed to examine ways that healthcare setting factors relate to clients’ perceived control over health-related decisions and behaviors [9, 10]. However, most health-related empowerment research has focused on chronic health conditions, such as mental health, diabetes, cancers, and disability [11–15], with less focus on women’s health-related empowerment, especially in low-resource settings.

Since maternal and child health services comprise women’s primary contact with the healthcare system in sub-Saharan Africa, it is important to understand how the delivery of these services reflects women’s values and sense of control over their own health. Pregnancy and antenatal care (ANC) are often the entry into the cascade of maternal and child health services, including prevention of maternal-to-child transmission of HIV, labor and delivery, postnatal services, contraception, and well-child care. During pregnancy, most women are essentially healthy and able to actively engage in their own healthcare. Women who report being empowered should be able to increase uptake of healthy behaviors, such as completing the recommended number of ANC or postnatal contacts. However, little is known about women’s empowerment during pregnancy or the factors that foster pregnancy-related empowerment.

Most countries in sub-Saharan Africa follow the World Health Organization’s recommendations and have adopted a four-contact model termed focused antenatal care (FANC) as the standard of care [16, 17]. FANC was designed to offer high-quality, intensive, and woman-centered ANC visits, now referred to as contacts, indicating the importance of an active connection between women and her provider [18, 19]. However, acute health worker shortages and underfunding prevent FANC from being implemented as intended [20, 21]. Properly conducting FANC should take 45 minutes for the first contact and 35 for follow-up contacts. However, an observational study in Tanzania documented that the average first contact lasted 12 minutes and follow-up contacts lasted only 7 minutes [22]. Moreover, health workers did not provide all recommended services [23] and were often disrespectful [24–26]. Perhaps reflecting the poor quality of services, many women do not complete the recommended number of contacts [27].

To address some of these gaps, our team adapted and piloted a model of group ANC based on CenteringPregnancy® (CP) for use in the two sub-Saharan African countries where our team had prior research experience, Malawi and Tanzania [28–30]. CP integrates three important dimensions of woman-centered care, namely healthcare in group space, interactive learning, and community building [31]. Its efficacy has been well documented in the US [32–36]. In CP, the same group of 8–12 women meet with the same providers in 2-hour ANC contacts throughout pregnancy. One provider can serve 12 clients in 120 minutes. This is similar to the length of observed individual FANC contacts, but group contacts provide each woman with up to 90 minutes of interactive discussions.

To examine the impact of individual ANC versus group ANC on women’s empowerment during pregnancy, we needed a measure of pregnancy-related empowerment. At present, only one scale exists [37]. The Pregnancy-Related Empowerment Scale (PRES) evaluates the quality of communication and connectedness pregnant women feel with their care providers and peers, their participation in decision-making, and their capacity to recognize and engage in pregnancy-related healthy behaviors. The PRES builds upon the concept of health-related empowerment and integrates social theory [38], feminist theory [39], and Bandura’s theory of self-efficacy [40, 41]. The PRES was validated as a tool to measure empowerment among low-income pregnant African American and Hispanic women in the U.S., but it has not been used in sub-Saharan Africa.

The purpose of this paper was to compare pregnancy-related empowerment, as measured by PRES scores, for women who attended individual ANC and CP-based group ANC at clinics in Malawi and Tanzania. We expected women in group ANC to have higher PRES scores because group care builds self-care skills, and offers continuity of care, more health promotion, and more contact time with providers and other women [31]. For each country, we examined the relationship between type of ANC and PRES scores, controlling for eight sociodemographic factors.

## Methods

### Design

This two-arm randomized controlled pilot study compared PRES scores for those assigned to individual ANC or CP-based group ANC (group ANC) at sites in rural Malawi and urban Tanzania.

### Setting and sample

Malawi and Tanzania are both low-income countries with high rates of maternal and infant mortality, but Malawi is substantially poorer and a larger proportion of its population is rural [42–44]. The study site located in central Malawi offered group ANC at two clinics, one located at a rural hospital and the other a rural health centre. The Tanzanian site included one clinic located in the city center of Dar es Salaam.

Between August and November of 2014, pregnant women who were 20–24 weeks pregnant, over age 16 and capable of completing study procedures were recruited and assessed for eligibility to participate. ANC midwives informed pregnant women that a research project was being conducted and, if the women were interested, the midwife escorted them to a private space in the clinic where research staff provided further information about the study. A total of 223 pregnant women were assessed for eligibility and 218 women provided consent. Baseline surveys, translated to Chichewa (Malawi) and Swahili (Tanzania), were administered to women using a touch screen computer and customized Computer-Assisted Personal Interview software developed at Tufts University [45]. After completing the baseline survey, a research assistant brought a basket filled with envelopes with equal numbers of assignments (1:1 ratio) to each study condition into the room. Women chose and opened one sealed envelope. Study condition assignment was concealed to research staff and women until the envelope was opened and the enclosed card was read aloud.

### Study conditions

#### Individual ANC (standard of care)

Women enrolled in individual ANC received FANC, the standard of practice in both countries. Women arriving to the antenatal clinic are served on a first come, first served basis. At some point in the day as women wait for services, they are assembled in the waiting area so that a midwife can deliver a health lecture on a predetermined topic. Where available, women complete laboratory tests and are encouraged to get an HIV test. Women have a one-on-one physical assessment and discussion with a midwife in a private room. The midwife (or an assistant) weighs her and takes and records her vitals. The expected number of ANC contacts is four and she may or may not see the same midwife over the course of the four contacts.

#### Group ANC (intervention)

For women enrolled in group ANC, the same midwife and co-facilitator provide 2 hours of care, education, and support for 12 women at each of the scheduled group ANC contacts. In Tanzania, women first go to the lab for services and then go to the group space. In Malawi, women do not routinely receive lab services, so they go directly to the group space upon arrival. In addition to socializing with group members, women participate in self-care activities by measuring and recording their own weight and vital signs. Each woman then has a 3- to 5-minute private meeting with the midwife in a corner of the group space. They discuss her health data and personal problems and the midwife conducts a physical assessment. If further examination is needed it is usually provided after the session ends. If a woman expresses a general concern or problem, the midwife will suggest that this be shared and discussed with the entire group either by the woman or by the midwife. After individual assessments are complete, the midwife and co-facilitator join the circle of women and facilitate interactive discussions using pre-arranged activities. Each session is appropriate for gestational age, but the discussion is fluid; women can bring up additional topics and the time allotted can change by degree of engagement.

### Measures

#### Dependent variable

The PRES is a 16-item Likert-type scale used to assess women’s sense of control over their pregnancy-related health and healthcare. Responses for each item ranged from 1 (strongly disagree) to 4 (strongly agree); the scale has a maximum score of 64. Scale development and content validity, as well as reliability for a sample of pregnant women in the USA, are described by Klima et al. [37].

#### Independent variables

Type of care (individual ANC or group ANC), was the primary independent variable for this study. Both Malawi and Tanzania administer ANC following FANC guidelines.

Based on established associations with pregnancy experiences and outcomes, we examined several sociodemographic factors. Age was divided into three groups (<20, 20–34, 35+) since adolescents and older mothers have different risks [46, 47]. Other variables included gravidity (primigravida or multigravida), religion (Muslim or Christian), and four indicators of socioeconomic status. Education was categorized into three categories (less than primary school, primary school completion, and more than primary school). We also looked at whether the woman said she was a subsistence farmer, indicative of a more rural lifestyle. We assessed extreme poverty using a single question regarding food insecurity – whether the woman had experienced lack of food or money to buy food in the past four weeks. To obtain some sense of the other end of the economic spectrum in terms of disposable income, we constructed an 10-item assets index that reflected how many of these items were owned [48].

### Procedure

Prior to data collection we received approvals from three institutional review boards – the University of Illinois at Chicago, the College of Medicine Research and Ethics Committee in Malawi, and the National Institute for Medical Research in Tanzania. We also received approval from the Ministries of Health and administrators at participating sites. We recruited participants, obtained informed consent and conducted the baseline survey. Women either attended individual ANC or group ANC throughout their pregnancy. The late pregnancy interview and PRES tool were scheduled to take place after the woman’s fourth ANC contact (between 32 and 38 weeks). When possible, the project manager made reminder phone calls. This strategy worked well in Tanzania, where over 95% of women had access to a cell phone, but was less successful in Malawi, where only 34% had access to a phone. Another strategy was meeting women at the clinic. For women in group ANC, the country project managers knew when the last group contact was scheduled, and thus made arrangements for the interviewers to be present at the clinic on those days. All interviews were conducted using the same in-person interview procedures for both individual and group participants. Extensive training of interviewers and use of the Computer-Assisted Personal Interview minimized potential interviewer bias.

### Analyses

Analyses were conducted separately for Malawi and Tanzania because baseline characteristics were significantly different for the two countries. We examined baseline sociodemographic factors of the study participants by study condition. Independent samples *t*-tests and Wilcoxon rank sums tests were employed to assess if mean PRES scores differed by study condition. In addition, we investigated sociodemographic factors to identify additional characteristics associated with pregnancy-related empowerment using model selection that maximized adjusted R-squared considering all subsets of predictors. Among the models selected, we tested moderation of treatment effect by the other variables in the models using two-way interaction terms.

In our primary analyses, participants with missing data were excluded through list-wise deletion. However, since differential retention occurred in Malawi, we compared our primary results to models estimated using the full information maximum likelihood approach to handling missing data. This approach is known to produce less biased estimates than complete case analyses [49, 50]. Using Mplus version 7 [51], we incorporated access to a cell phone, which was related to missingness, as an auxiliary variable in these inclusive, full information maximum likelihood models.

All regression analyses, *t*-tests, χ^2^ tests, and correlations were conducted using version SAS 9.4. Level of significance was set at *P* < 0.05 throughout; because of the small sample size, we also discussed trends (*P* < 0.10).

## Results

**Fig. 1:**
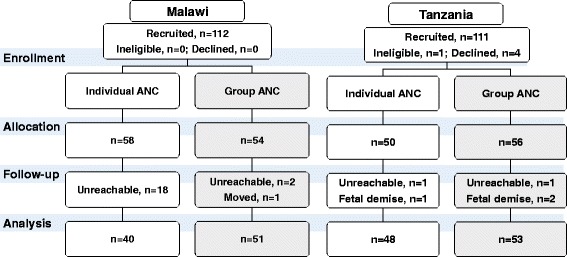
Participant flow for Malawi (left) and Tanzania (right)

Figure 1 shows the participant flow for each country. In Malawi, 58 women were allocated to individual ANC and 54 to group ANC, and in Tanzania 54 to individual ANC and 56 to group ANC. Retention was higher for women in group ANC than individual ANC in Malawi (94.4% vs. 69.0%, *P* = 0.0006) but similar in Tanzania (94.6% vs. 96.0%, *P* = 0.7421).

Baseline sociodemographic factors are presented in Table 1. In Malawi, one factor was different – by chance, more primigravid women were allocated to individual ANC than group ANC (41.4% vs. 18.9%, *P* < 0.013). In Tanzania, there were no sociodemographic differences by study condition. Thus, as would be expected with random assignment, the individual ANC and group ANC groups were highly equivalent at baseline. Table 1 also highlights the differences between countries. Regardless of group assignment, in Tanzania, about half the women were Muslim, while only one woman in Malawi was Muslim. Women in Tanzania also had more education, were less likely to be farmers, and had more food security and family assets.

**Table 1:** Participant baseline sociodemographic factors

	Full sample (*n* = 218)	Malawi (*n* = 112)		Tanzania (*n* = 106)	
		Individual ANC (*n* = 58)	Group ANC (*n* = 54)	Individual ANC (*n* = 50)	Group ANC (*n* = 56)
	*n* (%)	*n* (%)	*n* (%)	*n* (%)	*n* (%)
Age					
< 20	29 (13.5)	14 (24.6)	7 (13.2)	2 (4.1)	6 (10.9)
20 – 34	152 (71.0)	39 (68.4)	37 (69.8)	36 (73.5)	40 (72.7)
35 +	33 (15.4)	4 (7.0)	9 (17.0)	11 (22.4)	9 (16.4)
Gravidity					
Primigravid	67 (31.2)	24 (41.4)	10 (18.9)	16 (32.6)	17 (30.9)
Multigravid	148 (68.8)	34 (58.6)	43 (81.1)	33 (67.3)	38 (69.1)
Relationship					
Partner	197 (91.6)	57 (98.3)	52 (98.1)	45 (91.8)	43 (78.2)
Single	18 (8.4)	1 (1.7)	1 (1.9)	4 (8.2)	12 (21.8)
Religion					
Christian	164 (76.3)	57 (98.3)	53 (100.0)	27 (55.1)	27 (49.1)
Muslim	51 (23.7)	1 (1.72)	0 (0.0)	22 (44.9)	28 (50.9)
Education					
Less than primary	75 (34.9)	37 (63.8)	31 (58.5)	2 (4.1)	5 (9.1)
Primary	86 (40.0)	18 (31.0)	19 (35.8)	26 (53.1)	23 (41.8)
More than primary	54 (25.1)	3 (5.2)	3 (5.7)	21 (42.9)	27 (49.1)
Occupation					
Farmer	118 (54.9)	57 (98.3)	51 (96.2)	4 (8.2)	6 (10.9)
Other	97 (45.1)	1 (1.7)	2 (3.8)	45 (91.8)	49 (89.1)
Food secure					
Yes	153 (72.5)	39 (67.2)	33 (62.3)	36 (80.0)	45 (81.8)
No	58 (27.5)	19 (32.8)	20 (37.7)	9 (20.0)	10 (18.2)
	$\overline{\mathrm{x}}$ (SD)	$\overline{\mathrm{x}}$ (SD)	$\overline{\mathrm{x}}$ (SD)	$\overline{\mathrm{x}}$ (SD)	$\overline{\mathrm{x}}$ (SD)
Assets (0–10)	3.8 (2.1)	2.5 (1.2)	2.5 (1.3)	5.4 (1.7)	5.0 (2.0)

Individual PRES items for each country are in Table 2. In Malawi, every item was significantly different for individual and group ANC. In Tanzania, three items were significantly different, all of which were related to provider connectedness – having enough time with the midwife, if the midwife listens, and if the woman felt respected by her midwife.

**Table 2:** PRES item means (SD), by country and type of antenatal care

	Malawi		Tanzania	
	Individual ANC	Group ANC	Individual ANC	Group ANC
Provider Connectedness				
I can ask my midwife provider about my pregnancy	2.83 (0.5)	3.80 (0.4)*	3.19 (0.4)	3.30 (0.5)
I have enough time with my midwife to discuss my pregnancy	2.60 (0.6)	3.69 (0.5)*	3.02 (0.5)	3.28 (0.5)*
My midwife listens to me	2.54 (0.6)	3.71 (0.5)*	3.15 (0.4)	3.34 (0.5)*
My midwife respects me	2.70 (0.5)	3.71 (0.5)*	3.15 (0.4)	3.34 (0.5)*
I expect my midwife to respect my decisions about my pregnancy	2.67 (0.5)	3.71 (0.5)*	3.15 (0.4)	3.25 (0.5)
My midwife respects my decision, even if it is different than their recommendation	2.58 (0.5)	3.61 (0.5)*	3.0 (0.7)	2.85 (0.8)
Skillful Decision-Making				
I take responsibility for the decisions I make about my pregnancy like eating healthy food	2.98 (0.4)	3.80 (0.4)*	2.46 (0.9)	2.57 (0.9)
I can tell when I have made a good health choice	2.85 (0.4)	3.69 (0.5)*	3.21 (0.5)	3.21 (0.4)
Since I began prenatal care, I have been making more decisions about my health	2.85 (0.5)	3.67 (0.5)*	3.21 (0.4)	3.26 (0.4)
Peer Connectedness				
Women need to share experiences with other women when they are pregnant	2.83 (0.7)	3.69 (0.5)*	3.23 (0.4)	3.28 (0.5)
I share my feelings and experiences with other women	2.70 (0.6)	3.65 (0.5)*	3.15 (0.5)	3.23 (0.4)
Gaining Voice				
I know if I am gaining the right amount of weight during my pregnancy	2.40 (0.6)	3.67 (0.5)*	3.15 (0.5)	3.26 (0.4)
I have a right to ask questions when I don’t understand something about my pregnancy	2.50 (0.7)	3.69 (0.5)*	3.27 (0.4)	3.32 (0.5)
I am able to change things in my life that are not healthy for me	2.78 (0.5)	3.67 (0.5)*	3.19 (0.5)	3.26 (0.5)
I am doing what I can to have a healthy baby	2.97 (0.5)	3.67 (0.5)*	3.23 (0.5)	3.30 (0.5)
If something is going wrong in my pregnancy, I know who to talk to	2.97 (0.5)	3.69 (0.5)*	3.25 (0.4)	3.28 (0.5)

*Significantly different at *P* < 0.05

Table 3 shows the results of the independent samples *t*-tests for PRES by type of care. In Malawi, we found that the women receiving group ANC scored higher, on average, than those receiving individual care, whereas in Tanzania, this difference was much smaller and not statistically significant.

**Table 3:** Independent samples *t*-tests for PRES by type of care

	PRES Mean (SD)		*P* value
	Individual ANC	Group ANC	
Malawi	43.7 (4.5) *n* = 40	59.1 (5.9) *n* = 51	< 0.0001
Tanzania	50.0 (6.4) *n* = 48	51.4 (7.1) *n* = 53	0.305

The results of the multiple linear regression models selected to maximize the adjusted R-square from among the eight sociodemographic variables are listed in Table 4. In Malawi, type of care was the strongest statistically significant predictor in a model that accounted for 67% of the variance of PRES scores. Other variables in this model, though not statistically significant, suggested women over the age of 35 were more empowered than women aged 20–35 and women with recent food insecurity were less empowered. Two-way interactions for type of care with other predictors were not significant and were removed from the model. To address concerns about differential retention by study condition in Malawi, we analyzed all 112 participants enrolled in the Malawi study with full information maximum likelihood models using access to a cell phone as an auxiliary variable for its relation to missingness. The type of care model as well as the predictive model showed similar parameter estimates and was consistent with the statistical conclusions presented in Table 4.

**Table 4:** Predictive multiple linear regression models of PRES for each country

Malawi (*n* = 90)		
Variable	β(se)*	P value
Type of care (1 = group ANC, 0 = individual ANC)	15.29 (1.16)	< 0.0001
Age		
Age 20–35	REF	–
Age < 20	0.81 (1.42)	
Age > 35	2.42 (1.71)	0.160
Food insecurity	–1.44 (1.21)	0.236
Adjusted R-square	0.67	
Tanzania (*n* = 99)		
Variable	β (se)	P value
Type of care (1 = group ANC, 0 = individual ANC)	1.843 (1.34)	0.172
Religion (1 = Muslim, 0 = Christian)	–5.07 (1.92)	0.010
Type of care*Religion	5.02 (2.65)	0.061
Gravidity (1 = multigravida, 0 = primigravida)	2.17 (1.44)	0.135
Adjusted R-square	0.07	

*β(se): unstandardized regression coefficient and standard error

In Tanzania, the predictive model included type of care, religion, and gravidity. We examined two-way interactions of type of care with the other variables, retaining the marginally significant type of care by religion interaction. This model shows that Muslim women had lower empowerment than Christian women, except when receiving group care, in which case their empowerment level was similar to Christian women. In addition, though not statistically significant, group care and having a previous pregnancy were associated with greater pregnancy-related empowerment controlling for all variables in the model. This model predicted only 7% of the variability in PRES scores in Tanzania. Due to the findings of this predictive model, we examined the type of care effect on PRES stratified by religion using Wilcoxon rank sums two-sample tests. Muslim women in group ANC had higher mean PRES compared to individual care (mean (SD) group ANC, 51.3 (7.3); individual ANC, 47.1 (3.5); Wilcoxon = 421.5, *P* = 0.0153) whereas group and individual care were not significantly different among Christian women (group ANC, 51.7(7.0); individual ANC, 52.2 (7.4); Wilcoxon = 651.0, *P* = 0.9921).

## Discussion

To the best of our knowledge, this is the first study to use the PRES outside of the USA. For each country, the scale had good internal consistency reliability (α > 0.95).

CP-based group ANC is designed to provide continuity of care, build self-care skills, and ensure the forming of connections with providers as well as other pregnant women in the group [31]. Community building occurs as women and providers get to know one another and explore shared experiences. Therefore, we expected that participation in group ANC would result in higher levels of pregnancy-related empowerment.

This expectation was only partially confirmed in this study. Group ANC was strongly related to higher pregnancy-related empowerment in Malawi. In Tanzania, overall pregnancy-related empowerment did not differ by type of care. However, among Muslim women, group ANC was associated with significantly higher pregnancy-related empowerment, but type of care was not associated with pregnancy-related empowerment among Christian women.

Given our small sample and the lack of both urban and rural sites in each country, identification of possible factors contributing to these country differences is speculative. One factor that needs more exploration is whether women in urban settings have access to a wider range of opportunities and are, in general, more empowered prior to pregnancy [52]. However, if this were the case, group ANC should have the same effect for all urban women. Instead, Muslim women in Tanzania had slightly higher PRES scores if they were in group ANC. Muslim women in this sample were younger and reported more food insecurity, suggesting that they are socioeconomically disadvantaged compared to Christian women in this sa﻿mple. Lower levels of empowerment in health-related decision-making among Muslims have been reported in other African countries [53–55]. These findings are also congruent with USA studies showing that CP group ANC had greater benefits among more marginalized groups of women [56, 57].

A second possible factor may be related to perception of quality of care. Group ANC may not have as great an effect on pregnancy-related empowerment in clinics where individual care is perceived as high quality. The site in Dar es Salaam has a reputation for being one of the better government facilities in the city and serves a population of emp﻿loyed women. Since women make decisions about health services based on perceived quality [58], the women who chose to come to this clinic may have already had higher pregnancy-related empowerment.

A third factor may be related to the length of group sessions. Women in group ANC in Malawi received nearly twice as much contact time per session as women in Tanzania, where sessions lasted approximately 2 hours. In Malawi, the facilitators implemented the model with flexibility and allowed sessions to continue until all issues were discussed. Although this made the sessions longer than intended, the longer sessions may have contributed to greater pregnancy-related empowerment. In the context of four recommended antenatal contacts in both countries, ANC clients might benefit from the additional discussion time, either as longer sessions or more contacts, especially since the number of ANC contacts is considerably higher in most high-income countries [59, 60]. Recently, the World Health Organization has reevaluated ANC recommendations and supports increasing the total number of contacts to eight [19]. The issue of the optimal number of ANC contacts and total contact time during pregnancy certainly requires additional research.

### Limitations

A major limitation of this pilot was the lack of comparable urban and rural sites in both countries. We had initially planned to have four sites, one rural and one urban in each country; however, funding constraints forced us to limit the study. We felt it was important to examine whether group ANC could be implemented successfully in both rural and urban settings. Large urban metropolises, such as Dar es Salaam in Tanzania, offer many unique challenges. We also wanted to examine whether group ANC could work in settings with severely limited resources for both the health system and pregnant women. The substantially greater national poverty in Malawi made it an ideal setting for the study. This design provided strong evidence regarding the robustness of the CP-based ANC model in two very different settings. Because of our design choices, we are unable to disentangle the urban-rural and country differences. A larger randomized controlled study will allow for exploration of these issues.

A second limitation is that we only collected PRES data once in pregnancy. Because the PRES focused on ANC experiences, it was not appropriate to ask these questions at baseline. However, measurement of pregnancy-related empowerment at multiple time points in pregnancy would allow examination of whether PRES changes over the course of pregnancy and whether these changes are related to type of care.

Finally, differences between groups due to attrition bias are another potential limitation. However, the two approaches we used to examine the impact of missing data suggested that it had minimal effect on the results of this study.

## Conclusion and implications

This study provides evidence that ANC models affect pregnancy-related empowerment in some contexts. These pilot results indicate that, in a rural setting in Malawi where poverty is high, a CP-based group ANC model was associated with higher levels of pregnancy empowerment. However, in an urban setting in Tanzania, the same group ANC model was only related to higher pregnancy-related empowerment among Muslim women.

Education, socioeconomic status, parity, and partner status minimally related to pregnancy-related empowerment in this study. These results suggest that pregnancy-related empowerment is a distinct psychosocial phenomenon that does not simply mirror common sociodemographic factors. Moreover, changing the ANC model of care can have an impact on pregnancy-related empowerment in some contexts.

Pregnancy-related empowerment is important in sub-Saharan Africa, where low quality ANC and severe health worker shortages contribute to poor maternal and infant outcomes [61–63]. CP-based group ANC is a promising model to address these challenges and to increase pregnancy-related empowerment for some women. More research is needed to identify the ways that models of ANC can affect feelings of empowerment and perinatal outcomes for pregnant women globally [64].

### Open peer review

Peer review reports for this article are available in Additional file 1.

## Additional file

Additional file 1: Open peer review. (PDF 415 kb)

## Abbreviations

ANC: antenatal care
CP: CenteringPregnancy
EBF: exclusive breastfeeding
FANC: focused antenatal care
PRES: pregnancy-related empowerment scale
SD: standard deviation
WHO: World Health Organization
