# Supplementary material for: Randomized controlled pilot of a group antenatal care model and the sociodemographic factors associated with pregnancy-related empowerment in sub-Saharan Africa
Source: BMC Pregnancy Childbirth. 2017 Nov 8;17(Suppl 2):336. doi: 10.1186/s12884-017-1493-3 (PMC5688418; doi:10.1186/s12884-017-1493-3)
Supplement: Additional file 1: — Open peer review. (PDF 415 kb) [file 12884_2017_1493_MOESM1_ESM.pdf]

Reviewer reports

Title: Randomized controlled pilot of a group antenatal care model and the sociodemographic factors associated with pregnancy-related empowerment in sub-Saharan Africa

Reviewer 1: Meg Autry

- Major Compulsory Revisions

1. This is really an observational study that looks at a centering pregnancy model in two countries in two different settings. You cannot directly compare as two different countries and one is rural and one is urban. You need to eliminate all direct comparisons and merely state the findings of the individual groups/settings.
2. Your paper is entirely too long and convoluted which takes away from its excellent message. I would significantly shorten it and eliminate some of the tables that you describe in the article. Your conclusions are that in a rural setting in Malawi where education low and poverty high, a centering pregnancy model improves pregnancy empowerment. In an urban setting in Tanzania, a group pregnancy model empowers only in young Muslim women.
2. Some of your statements are too strong throughout the article – "never evaluated before", "never studied before". I would soften these up to something like "to our knowledge, never studied before", "extensive literature review reveals no prior studies"
3. You never define the acronym FANC

- Minor Essential Revisions

line 317 – conducted is spelled wrong.

- Discretionary Revisions
Elimination of some of the tables
Simplifying the article by making the conclusions stated above and eliminate the models

Level of interest - An article of importance in its field

Quality of written English - Acceptable

I declare that I have no competing interests.

Reviewer 2: Joelle Brown

1. Is the question posed by the authors well defined?
The authors seek to understand the impact of group vs standard antenatal care counseling on pregnancy empowerment. The research question posed by the authors is well defined, thought the timing of the empowerment outcome is unclear.

2. Are the methods appropriate and well described?

This was an unblinded randomized controlled pilot study assessing the impact of a novel group antenatal care model (4 group antenatal care visits over the duration of pregnancy) vs. standard of care (4 individual antenatal care visits over the duration of pregnancy) on pregnancy related empowerment among women in Tanzania and Malawi. Pregnancy related empowerment was estimated using a novel, validated pregnancy-related empowerment scale.

Overall the methods were well described. However, I have a few major concerns (see below) and outline issues that must be addressed before publication is considered.

3. Are the data sound?
In general, the data appear sound. However, I have a few major comments/suggestions (see below).

4. Do the figures appear to be genuine, i.e. without evidence of manipulation?
The figures appear to be genuine

5. Does the manuscript adhere to the relevant standards for reporting and data deposition?
The manuscript appears to adhere to the relevant standards for reporting. There is no mention of data deposition.

6. Are the discussion and conclusions well balanced and adequately supported by the data?'

In general, the discussion and conclusions are well balances and adequately supported by the data. However, additional explanation is needed in the last section of the Discussion.

7. Are limitations of the work clearly stated?
Some limitations are discussed. However there are additional limitations that need inclusion, specifically, no measurement of baseline empowerment, high and differential loss to follow-up, no measurement of biological outcomes, and possibly others (as discussed above).

8. Do the authors clearly acknowledge any work upon which they are building, both published and unpublished?
The authors appear to clearly acknowledge work upon which they are building.

9. Do the title and abstract accurately convey what has been found?
The title and abstract do convey what has been found. Although I would suggest that the study design (randomized controlled trial) be included in the title.

10. Is the writing acceptable?
In general the writing is acceptable. However, the manuscript could benefit from additional copy editing. For example, some of the methods section is written in the past tense and some in the future tense. Figure 1 is not well described.

- Major Compulsory Revisions

1. Please clarify whether/why were there no baseline measure of pregnancy related empowerment prior to the intervention? Looking at the average within woman change in pregnancy related empowerment by randomization group would have provided very important information about the impact of the intervention in both settings.

Also, given the significant loss to follow-up during the study (see below) a baseline measure of PRES would have allowed the authors to evaluate whether women who were less empowered to begin with were more likely to drop out of ANC care.

If no baseline PRES measurement was made then this should be discussed as a limitation.

2. Please include in the methods a discussion of 1) how many times outcome PRES scores were collected and at 2) which visit(s)/months(s), 3) how the outcome data were collected (interviewer assisted, self administered, ACASI, individual interviews, group interviews, etc), 4) how interviewer bias was minimized in the collection of outcome data, and 5) explain whether the outcome data were collected in the same way for participants in the intervention and control group.

Also please include a discussion or when/after how many visits the authors expected empowerment to occur during the intervention.

3. Why was there such a difference in implementation of the intervention at the two sites? Can the authors discuss these differences more and suggest whether they would advocate for flexibility in implementation, longer counseling sessions, etc during the administration of the intervention?

A stronger study design would have included urban and rural sites within each country. This should be included as a limitation.

4. There were such significant differences in the study sites in Malawi and Tanzania (urban vs rural) the implementation of the intervention at each site (4 hours per visit vs 2 hours per visit),

the participants (age, religion, relationship status, SES, etc) in each site, and the outcome in each site (intervention effective in Malawi, not effective in Tanzania). Why did the authors choose to combine the data rather than analyse each country dataset separately?

5. In the Methods section, paragraph 1 Study Design, the authors state "This analysis examined the relationship of type of care to several obstetric, socioeconomic and cultural variables at baseline…" It does not make sense to examine the impact of the intervention on baseline variables. Please clarify. Also, please clarify whether it will be possible to measure the effect of the intervention on uptake of ANC services, pregnancy outcomes, or birth outcomes.

6. Methods section, paragraph 3 Setting and Sample: the authors state that 218 women were recruited, but only 104 were randomized to intervention and 88 to control (n=192). What happened to the other 26 women?

Please check the text of the manuscript and be consistent with the sample size throughout.

7. Methods section, paragraph 4: Approximately 25% of women in the control group, and 13% of women in the intervention group were lost to follow-up. What attempts were made to contact women who failed to return for follow-up ANC visits, or to measure pregnancy related empowerment outcomes, and track their birth outcomes? Please can the authors discuss how this significant loss to follow-up could have led to bias in their results and ultimately their interpretation of the effectiveness of the CenteringPregnancy tool. And were women in one country more likely to be lost to follow-up?

Also, please clarify how it is possible that the overall retention is cited as 88% while the retention in the intervention group is 87.3% and the retention in the control group is 74.1%.

8. Do the authors think that one possible unintended benefit of CenteringPregnancy is higher retention in women receiving group antenatal care counseling?

9. In Model 4 of the analysis, how were variables chosen for inclusion in the final model?

10. Please clarify if the randomization assignment was 1:1, and if so, why the sample size in the intervention group is 104 while the sample size in the control group is 88.

11. In Figure 1, what variables were used to create the adjusted estimates? Please make the Figure 1 self explanatory.

12. Please provide results on the average length of time the sessions took, and the range. Also, please provide the average number of sessions each woman attended, IQR and range.

13. Table 4 was not included in the manuscript. Please add.

14. Table 1 is a summary of baseline characteristics. Is the PRES score in Table 1 collected at the baseline visit or at a follow-up visit?

15. Was the intervention equally effective in all age groups? All SES groups? In single women?

16. Were exact statistical methods used for small cells in Table 1?

17. The following statement in the Discussion needs further explanation: “The study provides evidence that pregnancy related empowerment is a distinct concept that is not interchangeable with obstetric, socioeconomic, and cultural indicators.” How does this study show that?

- Minor Essential Revisions

1. Methods section, paragraph 2 Setting and Sample: please clarify in which cities and centres the study took place.

2. In the last paragraph of the Measures section in the Methods, please provide the reference for the standard assets index.

3. Please be consistent throughout the manuscript with the use of terminology referring to the two study groups.

4. The last paragraph of the Introduction seems out of place. Those details could be included under Methods/Setting and sample, or in the Discussion as the authors attempt to explain why the intervention appears to increase empowerment in Malawi, but not in Tanzania.

5. The manuscript is well written, but could benefit from copy editing. For example, some of the methods section is written in the past tense and some in the future tense.

Level of interest - An article whose findings are important to those with closely related research interests
Quality of written English - Needs some language corrections before being published

I declare that I have no competing interests.

Response to reviewers

Reviewers,
Thank you for your thoughtful review of this manuscript. We have substantially revised it based on your insights. We appreciate the attention to detail and hope that we have addressed all of your concerns in this revised manuscript. Below we address the major issue raised by all three reviewers. This is followed by responses to individual reviewer statements.

Major issue: Whether to eliminate the total sample comparison:
The Managing Editor and one reviewer argued that the paper should only present analyses separately by country, and that the analyses for the total sample should be dropped. The second reviewer (Joelle Brown) asked for an explanation of why we analyzed the data together instead of analyzing for each country separately.

Response: We believe there is merit in retaining analyses for the total data set as well as separate analyses by country. This analytic approach accurately reflects the study as designed and implemented. Regressions using the total sample also demonstrates that pregnancy-related empowerment did not relate to any of the obstetric and sociodemographic factors we included except for religion. Such a lack of impact from factors such as age, education, parity and several indicators of economic status is an important and distinctive finding. This paper examines one outcome for a small randomized clinical trial pilot that includes multiple perinatal outcomes that will be published in later manuscripts. For this specific outcome of pregnancy-related empowerment, group ANC had a positive impact in one country but not the other. However, for all other outcomes that we have examined to date, there was a consistent similar impact across both countries. We also would like to retain a consistent analytic approach across manuscripts.

We have shortened and simplified the total sample presentation. We have accepted Reviewer 1's suggestion that we do not include all four steps of the regression for the total sample. With corresponding shortening of the analysis description, this approach allowed us to combine Tables 3 and 4 into a single Table, a new Table 3.We also want to clarify that the submission included a full analysis of each country separately as well as analysis using the total dataset. There was an error in the assembly of the pdf, it did not contain Table 4 (the separate regression analyses for each country now part of new Table 3).

In the table below we address comments that were more unique to each reviewer:

Comments:	Responses
**Comments from Managing Editor, Paula Tavrow**	
In addition to making the revisions requested of the reviewers, the managing editor believes that the paper would benefit from **a chart showing how many people completed the intervention from each country, who was lost to follow-up, as well as sensitivity analysis of what the loss to follow-up might have meant for the study results.** This is particularly important because there was no baseline of the PRES.	We include a diagram (Figure 1) showing participant flow through the study.<br><br>To examine the effects of differential loss to follow-up, GLM results were compared to models estimated using the full information maximum likelihood (FIML) approach to handling missing data, known to produce less biased estimates than complete case analyses (Graham, Enders) and a reasonable approach for data assumed missing at random (i.e., related to treatment group and cell phone ownership). Using Mplus version 7, we incorporated cell phone ownership, which was related to missingness, as an auxiliary variable in these inclusive FIML models. These FIML models were very similar to our GLM models. We report this approach in the manuscript. In addition, we conducted two sensitivity analyses using imputed high and low PRES scores for missing data for the Malawi sample. In fully adjusted models using these imputed data, our findings were robust for the effect of type of care (p<.0001 in both models). For the model with missing imputed as a low PRES score, the adjusted means for type of care were CP= 57.7, IC=43.3. For the model with missing imputed as a high PRES score, the adjusted means for type of care were CP= 59.4, IC=49.9. Thus, our sensitivity analyses showed stable statistical conclusions as well.
Lastly, while the subject of this paper was the extent to which the intervention seemed to increase PRES, one wonders if empowerment and/or group ANC were ultimately linked to pregnancy outcomes (e.g., delivering in a facility). If this information is available, it would be a valuable addition to the paper (unless it will be used in another paper).	We have a fairly extensive array of other perinatal outcomes, but these will be presented in other papers.
**Reviewer #1 Meg Autry**	
**Major Compulsory Revisions**	
This is really an observational study that looks at a centering pregnancy model in two countries in two different settings. You cannot directly compare as two different countries and one is rural and one is urban. You need to eliminate all direct comparisons and merely state the findings of the individual groups/settings.	See above. Please note that as stated by reviewer #2, this was an unblinded randomized controlled pilot study.
Your paper is entirely too long and convoluted which takes away from its excellent message. I would **significantly shorten it** and **eliminate some of the tables** that you describe in the article. Your conclusions are that in a rural setting in Malawi where education low and poverty high, a centering pregnancy model improves pregnancy empowerment. In an urban setting in Tanzania, a group pregnancy model empowers only in young Muslim women.	We have made several cuts to the text and eliminated one table. However, one figure was added at the request of the managing editor. We appreciate the summary statement and have integrated it into the manuscript.

Some of your statements are too strong throughout the article – “never evaluated before”, “never studied before”. I would soften these up to something like “to our knowledge, never studied before”, “extensive literature review reveals no prior studies”	We have changed these throughout.
You never define the acronym FANC	Corrected
**Minor Essential Revisions**	
Line 317 – conducted is spelled wrong.	Corrected
**Discretionary Revisions**	
Elimination of some of the tables	We eliminated one table
Simplifying the article by making the conclusions stated above and eliminate the models	We have eliminated presenting all 4 models for the total.
**Reviewer #2 Joelle Brown**	
The research question posed by the authors is well defined, though the timing of the empowerment outcome is unclear.	We now state that the late pregnancy interview was scheduled for 32-36 weeks gestation.
The manuscript appears to adhere to the relevant standards for reporting. There is no mention of data deposition.	Following NIH guidelines, de-identified data will become available upon request after analyses are completed.
In general, the discussion and conclusions are well balances and adequately supported by the data. However, additional explanation is needed in the last section of the Discussion.	We have added clarification, simplified and link conclusions to the data.
Some limitations are discussed. However there are additional limitations that need inclusion, specifically, no measurement of baseline empowerment, high and differential loss to follow-up, no measurement of biological outcomes, and possibly others (as discussed above).	We corrected loss to follow-up data. We (incorrectly) included those declining to consent or ineligible. Overall loss to follow-up was not high, however it was high for women in individual care in Malawi (see Figure 2). We imputed extreme high and low PRES scores for missing data using the full information maximum likelihood (FIML) approach to handling missing data. In fully adjusted models using these imputed data, our findings were robust for the effect of type of care ($p<.0001$ in both models). Other outcomes will be published in later manuscripts.
In general, the writing is acceptable. However, the manuscript could benefit from additional copy editing. For example, some of the methods section is written in the past tense and some in the future tense. Figure 1 is not well described.	We have made the methods consistent and added a fuller description of Figure 2 (formerly Figure 1).
**Major Compulsory Revisions**	
Please clarify whether/why were there no baseline measure of pregnancy related empowerment prior to the intervention? Looking at the average within woman change in pregnancy related empowerment by randomization group would have provided very important information about the impact of the intervention in both settings. Also, given the significant loss to follow-up during the study (see below) a baseline measure of PRES would have allowed the authors to evaluate whether women who were less empowered to begin with were more likely to drop out of ANC care.	The PRES is specific to the current pregnancy and the clinical experiences/services of ANC (see Table 2); therefore it would be inappropriate to assess this at baseline before women attended their first ANC visit. However, we do note in the discussion that more than more measure in pregnancy would be helpful in future studies.
If no baseline PRES measurement was made then this should be discussed as a limitation.	See above

2. Please include in the methods a discussion of 1) how many times outcome PRES scores were collected and at 2) which visit(s)/months(s), 3) how the outcome data were collected (interviewer assisted, self-administered, ACASI, individual interviews, group interviews, etc), 4) how interviewer bias was minimized in the collection of outcome data, and 5) explain whether the outcome data were collected in the same way for participants in the intervention and control group.	These points have all been added in methods under the dependent variable and procedures.
Also please include a discussion or when/after how many visits the authors expected empowerment to occur during the intervention.	Our expectation was that the PRES would improve gradually over the course of the group ANC intervention. We measured the outcome after the 4th ANC visit was to be completed.
Why was there such a difference in implementation of the intervention at the two sites? Can the authors discuss these differences more and suggest whether they would advocate for flexibility in implementation, longer counseling sessions, etc during the administration of the intervention?	Inadequate clarification of this parameter plus a situation in the rural Malawi site that allowed providers and women to stay longer – our recommendation is to either increase the time per session or increase the total number of ANC visits. It is difficult to state whether the longer sessions in fact contributed to higher empowerment in Malawi, especially since for most other outcomes (not presented here) this does not seem to matter.
A stronger study design would have included urban and rural sites within each country. This should be included as a limitation.	This has been added to limitations and recommendations for future studies. See above
There were such significant differences in the study sites in Malawi and Tanzania (urban vs rural) the implementation of the intervention at each site (4 hours per visit vs 2 hours per visit), the participants (age, religion, relationship status, SES, etc) in each site, and the outcome in each site (intervention effective in Malawi, not effective in Tanzania). Why did the authors choose to combine the data rather than analyze each country dataset separately?	See statement at the beginning of this response and other details provided above.
In the Methods section, paragraph 1 Study Design, the authors state "This analysis examined the relationship of type of care to several obstetric, socioeconomic and cultural variables at baseline…" It does not make sense to examine the impact of the intervention on baseline variables. Please clarify. Also, please clarify whether it will be possible to measure the effect of the intervention on uptake of ANC services, pregnancy outcomes, or birth outcomes.	Added. We examined the relationship between baseline characteristics and the intervention to see if the intervention had different impacts for women with different baseline characteristics. We do include other outcomes, which will be in future publications.
6. Methods section, paragraph 3 Setting and Sample: the authors state that 218 women were recruited, but only 104 were randomized to intervention and 88 to control (n=192). What happened to the other 26 women?	See figure 1
Please check the text of the manuscript and be consistent with the sample size throughout.	Done.

Methods section, paragraph 4: Approximately 25% of women in the control group, and 13% of women in the intervention group were lost to follow-up. What attempts were made to contact women who failed to return for follow-up ANC visits, or to measure pregnancy related empowerment outcomes, and track their birth outcomes? Please can the authors discuss how this significant loss to follow-up could have led to bias in their results and ultimately their interpretation of the effectiveness of the CenteringPregnancy tool. And were women in one country more likely to be lost to follow-up?	See correct lost to follow-up data and our discussion of this in the analysis and result (see above as well)
Also, please clarify how it is possible that the overall retention is cited as 88% while the retention in the intervention group is 87.3% and the retention in the control group is 74.1%.	Corrected. See figure 1
Do the authors think that one possible unintended benefit of CenteringPregnancy is higher retention in women receiving group antenatal care counseling?	Yes, it is true that women are more engaged in their care, it's more fun and takes less time. Other results to be reported elsewhere to document this.
In Model 4 of the analysis, how were variables chosen for inclusion in the final model?	Heather – I think this is already clear!
Please clarify if the randomization assignment was 1:1, and if so, why the sample size in the intervention group is 104 while the sample size in the control group is 88.	The randomization was nearly 1:1 (108 vs. 110). It was the differential loss to follow-up that led to this difference in sample size at the late pregnancy interview.
In Figure 1, what variables were used to create the adjusted estimates? Please make the Figure 1 self explanatory.	See Figure 2. Discuss
Please provide results on the average length of time the sessions took, and the range. Also, please provide the average number of sessions each woman attended, IQR and range.	We included mean and standard deviation. We did not yet calculate the number of visits IQR and range for the dataset. That will be published in a future paper on healthcare utilization impacts of group ANC.
13. Table 4 was not included in the manuscript. Please add.	We are sorry. Table 4, the separate analyses by country, was inadvertently omitted from the pdf submission. No wonder there was confusion! See the new Table 3 (we combined the original Tables 3 & 4 into a single new table).
Table 1 is a summary of baseline characteristics. Is the PRES score in Table 1 collected at the baseline visit or at a follow-up visit?	See new title for Table 1. Basic sociodemographic characteristics were measured at baseline and ANC-related data were collected in late pregnancy.
Was the intervention equally effective in all age groups? All SES groups? In single women?	The intervention was effective for all in Malawi. This did not hold for Tanzania with the exception of Muslim women.
Were exact statistical methods used for small cells in Table 1?	Yes.
The following statement in the Discussion needs further explanation: "The study provides evidence that pregnancy related empowerment is a distinct concept that is not interchangeable with obstetric, socioeconomic, and cultural indicators." How does this study show that?	Deleted. We mean to say that, none of those other characteristics related to the PRES scores in Malawi. In Tanzania, only 8% of variation is explained and its not
**Minor Essential Revisions**	
Methods section, paragraph 2 Setting and Sample: please clarify in which cities and centres the study took place.	As per our IRB, we are not naming the specific clinics.

In the last paragraph of the Measures section in the Methods, please provide the reference for the standard assets index.	Added.
Please be consistent throughout the manuscript with the use of terminology referring to the two study groups.	Individual ANC and Group ANC are the terms you will now see.
The last paragraph of the Introduction seems out of place. Those details could be included under Methods/Setting and sample, or in the Discussion as the authors attempt to explain why the intervention appears to increase empowerment in Malawi, but not in Tanzania.	We had toyed with putting it in the discussion. I shortened it but if we want to rework it for the discussion, I can work on that.
The manuscript is well written, but could benefit from copy editing. For example, some of the methods section is written in the past tense and some in the future tense.	We have worked to reduce the total number of pages and make our logic more succinct. Overall, we have cleaned up sentence structure and organization to improve readability.

Again, we thank you for taking the time to review our manuscript. We look forward to your response.

Sincerely,
Crystal L. Patil

Reviewer reports – 2nd round

Dear Dr. Crystal Patil,

12/9/2016

Thank you for your comments and revisions to the manuscript, "Randomized Controlled Pilot of a Group Antenatal Care Model and the Sociodemographic Factors Associated with Pregnancy-Related Empowerment in sub-Saharan Africa,"which you had submitted to the BMC Pregnancy and Childbirth Special Issue on Women's Health and Empowerment.

We sent the revised manuscript back to the two original reviewers. Both felt that the manuscript still requires major revision. The first reviewer (Autry) wrote her comments directly on the revised manuscript (attached). The other reviewer (Brown) continued to express serious reservations about the statistical analysis. To get another view, we hired a statistician (Telesca) to offer his expert opinion about the second reviewer's comments and also to give his own feedback. The second reviewer's comments and the statistician's appraisal are both attached. The statistician agreed with the second reviewer that the statistical analysis needs to be re-done.

After reviewing this second round of comments, the editors agree that your article requires further revisions before we can consider it for publication. Although we understand that you had wanted to combine the data from Malawi and Tanzania in your analysis, this does not seem to be an acceptable approach. Also, backward selection is not an advisable method of analysis because it lacks theoretical basis, so it would be better if you instead selected subsets of variables based on previous evidence or hypotheses. Lastly, some parts of the paper still are rather confusing and need to be rewritten, as per the reviewers' comments, including Table 1.

Given this feedback, we request that you submit a second revision of the manuscript for our review. Since the holidays are approaching and you may need to consult a statistician as you revise the paper, we have set a deadline of January 6, 2017, for these revisions, which is four weeks from today. This is the maximum amount of time we can give for the resubmission.

Please let us know if you will be able to make the revisions requested in the time allocated.

Best wishes for the holiday season.

Sincerely,
Paula Tavrow, PhD
Managing Editor, BMC Pregnancy and Childbirth Special Issue

Reviewer 1: Meg Autry

Name and email of primary contact: Crystal L. Patil, cpatil@uic.edu

Title of manuscript: Randomized Controlled Pilot of a Group Antenatal Care Model and the Sociodemographic Factors Associated with Pregnancy-Related Empowerment in sub-Saharan Africa

Authors: (full names and affiliations)

1 Molly McGown, Institute for Public Health and Medicine, Northwestern University, Chicago, IL, USA

2 Carrie S. Klima, Department of Women, Children and Family Health Science, College of Nursing, University of Illinois at Chicago, Chicago, IL, USA

3 Sebalda C. Leshabari, School of Nursing, Muhimbili University of Health and Allied Sciences, Dar es Salaam, Tanzania

4 Chrissie PN Kaponda, Nursing & Midwifery, Kamuzu College of Nursing, University of Malawi, Lilongwe, Malawi (currently Malawi High Commissioner for South Africa)

5 Alana D. Steffen, Department of Health Systems Science, University of Illinois at Chicago, Chicago, IL, USA

6 Heather Pauls, Office of Research Facilitation, University of Illinois at Chicago, Chicago, IL, USA

7 Willy Sangu, Ilala Municipality, Dar es Salaam, Tanzania

8 Kathleen F. Norr, Department of Women, Children and Family Health Science, College of Nursing, University of Illinois at Chicago, Chicago, IL, USA

9 Crystal L. Patil, Department of Women, Children and Family Health Science, College of Nursing, University of Illinois at Chicago, Chicago, IL, USA

Abstract

Background

The links between empowerment and a number of health-related outcomes in sub-Saharan Africa have been documented, but empowerment related to pregnancy is under-investigated. Antenatal care (ANC) is the entry point into the healthcare system for most women. It is important to understand how ANC affects women's sense of control over their health. This paper compares pregnancy-related empowerment for women in individual ANC (standard of care) versus CP-based group ANC (intervention) in Malawi and Tanzania.

Methods

Pregnant women in Malawi and Tanzania were recruited into a pilot study and randomized to CenteringPregnancy(CP)-based group ANC or individual ANC (n=218). Overall retention at late pregnancy was 88%. Both groups had four visits. In group ANC, each visit included self- and midwife-assessment in group space and 90 minutes of interactive health promotion. We measured pregnancy-related empowerment in late pregnancy using the Pregnancy-Related Empowerment Scale (PRES). General linear modeling (GLM) was employed to assess whether group ANC led to higher PRES scores than individual ANC, controlling for eight sociodemographic factors, for the total sample and by country. .

Results

The mean PRES score was 51.5 and was significantly higher for women in group ANC compared to individual ANC. In the final regression model for the entire sample, type of care, country and religion -were ~~was a~~ significant predictors of PRES. ~~along with country, religion, and a country by type of care interaction term; this explained 45% of the variation in PRES scores~~. In the final model for Malawi, type of care was the only significant predictor and explained 67% of the variation. In Tanzania, r~~egression only explained 8.5% of the variation in PRES scored~~. t~~T~~ype of care was not significant, but adjusted means for type of care by religion showed an interesting pattern with ~~. For Christian women, type of care was not related to PRES~~

Comment [AM1]: This should either be deleted in the abstract because it is confusing or explained further.

Comment [AM2]: Again, confusing and doesn't read well

~~scores. However,~~ m~~M~~uslim women in group ANC ha<u>ving</u>~~d~~ a higher mean PRES score than those in individual ANC.

Conclusions

Group antenatal care ~~is a promising model to~~ empower<u>s</u> pregnant women in some contexts while addressing the challenges of providing quality care despite low resources.

Keywords: Pregnancy-related empowerment - models of healthcare delivery -- antenatal care -- group ANC -- group care -- sub-Saharan Africa -- CenteringPregnancy®

Background

Empowerment is a complex multidimensional concept that can be broadly defined as the ability of individuals or groups "to improve capacities, to critically analyze situations and to take actions to improve those situations" [1]. In sub-Saharan Africa, women's overall empowerment has been positively associated with the utilization of maternal health services[2], use of contraception [3–5], improved infant feeding practices [6], and reductions in infant mortality [7].

Given these positive impacts of general empowerment on maternal-child health, it is important to consider women's empowerment as it relates specifically to health and health care [8]. Health-related empowerment assessment was developed to examine ways that healthcare setting factors relate to clients' perceived control over health-related decisions and behaviors [9,10]. However, most health-related empowerment research has focused on chronic health conditions, such as diabetes, cancers, mental health and disability [11–15]. Relatively little research has been conducted regarding women's health-related empowerment, especially in low-resource settings.

Since maternal and child health services comprise women's primary contact with the healthcare system in sub-Saharan Africa, it is important to understand how the delivery of these services affects women's sense of control over their own health. Pregnancy and antenatal care (ANC) are often the entry into the cascade of maternal and child health services, including prevention of maternal-to-child transmission of HIV, labor and delivery, postnatal services, contraception, and well-child care. During pregnancy most women are essentially healthy and able to actively engage in their own healthcare. When women have positive relationships with ANC providers and understand the rationale for recommended healthy pregnancy behaviors, they are likely to feel higher pregnancy-related empowerment.

In most countries of sub-Saharan Africa, the standard of care is a 4-visit model called Focused Antenatal Care (FANC) [16,17]. FANC is designed to offer high-quality, intensive, and woman-centered ANC visits [18]. However, acute health worker shortages and underfunding

Comment [AM3]: Or research?

Comment [AM4]: Although it is evident, I think there should be a statement about why empowerment is important – women who are more empowered are more likely to make important health decisions or take better care of their families or whatever?

Comment [AM5]: Is this the standard of care or is this was WHO recommends. Perhaps reword something like "While many strive to achieve the WHO recommended 4-visit model (FANC, acute health worker shortages and underfunding prevent...

prevent FANC from being implemented as intended [19,20]. Properly conducting FANC should take 45 minutes for the first visit and 35 for follow-up visits. However, an observational study in Tanzania documented that the average first visit lasted 12 minutes and follow-up visits lasted only 7 minutes [21]. Moreover, health workers did not provide all recommendedrequired services [22] and were often disrespectful [23–25]. Perhaps reflecting the poor quality of services, many women do not complete the recommended number of visits [26].

To address some of these gaps, our team adapted and piloted an alternative model of group ANC based on CenteringPregnancy® (CP) for use in the two countries in sub-Saharan Africa where our team had prior research experience, Malawi and Tanzania [27–29]. CP's efficacy has been well documented in the US. [30–35]. In CP, the same group of 8-12 women meet with the same providers in 2-hour ANC visits throughout pregnancy. One provider can serve 12 clients in 120 minutes, which averages to 10-minutes per woman [36], similar to the length of observed individual visits but with up to 90 minutes of focused discussion.

To examine the impact of group ANC versus individual ANC on women's empowerment during pregnancy, we needed a measure of pregnancy-related empowerment. At present, only one scale exists to measure pregnancy-related empowerment [37]. The Pregnancy-Related Empowerment Scale (PRES) evaluates the quality of communication and connectedness pregnant women feel with their care providers and peers, their participation in decision-making, and their capacity to recognize and engage in healthy behaviors. The PRES builds upon the concept of health-related empowerment and integrates social theory [38], feminist theory [39], and Bandura's theory of self-efficacy [10,40,41]. The PRES was validated as a tool to measure empowerment for low-income pregnant African American and Hispanic women in the U.S., but it has never been used in Africa [37].

The purpose of this paper was to compare pregnancy-related empowerment, as measured by PRES scores, for women who attended individual ANC (standard of care) and CP-based group ANC (intervention) clinics in Malawi and Tanzania. We expected that women in

group ANC would have higher PRES scores because group care offers continuity of care, building of self-care skills, more health promotion, and more contact time with providers and other women. We examined the relationship between type of care and PRES scores, controlling for eight sociodemographic factors~~,~~ for the total sample and within each country.

Methods

Design

This 2-arm randomized controlled pilot study compared PRES scores for those randomly assigned to individual ANC (standard of care) or CP-based group ANC (group ANC, intervention) at sites in rural Malawi and urban Tanzania.

Setting and Sample

The study was conducted in two sub-Saharan African countries, Malawi and Tanzania. One site was located in central Malawi at two different clinics: a District Hospital and one of its satellite clinics. The Tanzanian site included one clinic located in the city center of Dar es Salaam~~, a nascent mega city in Africa~~ [42,43]. Both countries are both low-income and have high rates of maternal and infant mortality, but Malawi is substantially poorer and more rural than Tanzania [44,45] .

Between August and November of 2014, pregnant women between 20-24 weeks pregnancy, over age 16 and capable of completing study procedures were eligible and recruited to participate. As shown in Figure 1, 223 pregnant women were assessed for eligibility and 218 women provided consent and completed the baseline survey. Each woman then selected a sealed envelope to randomly determine their study assignment; 108 were allocated to individual ANC and 110 to group ANC.

-- INSERT FIGURE 1 APPROXIMATELY HERE --

The overall retention rate for study participants from baseline to the late pregnancy interview was 88.1%. Retention was higher for women in group ANC than individual ANC (94.5% vs. 81.5%, p = 0.013) and in Tanzania than in Malawi (95.3% vs. 81.3%, p = 0.001).

Comment [AM6]: This highlighted section should be in results not methods

Formatted: Highlight

Formatted: Highlight

Formatted: Highlight

Nearly 70% of women lost to follow-up for the late pregnancy interview were in individual ANC in Malawi. Participants lost to follow-up were more likely to be Muslim ($p = 0.029$), have less education ($p < 0.001$) and fewer assets ($p < 0.001$). ~~Retention was most difficult for women in individual ANC in Malawi.~~ Compared to Tanzania where less than 5% lacked access to a cell phone, the majority of women in Malawi (66%) did not have access to a cell phone, likely reflecting that most were subsistence-level farmers and greater poverty levels in Malawi. ~~We~~ The~~scheduled the~~ late pregnancy interview was scheduled to coincide~~to correspond~~ with the woman's fourth ANC visit. For women in group ANC, the research team knew when their last group visit was scheduled, so the team arranged to interview them even if we could not reach them by cell phone.

Formatted: Highlight

Comment [AM7]: This highlighted section should be in discussion not results

Formatted: Highlight

Study Conditions

Individual ANC (standard of care)

Women enrolled in individual ANC typically arrive at the antenatal clinic and are served on a first come, first serve basis. While waiting for services, women assemble in a large waiting area where a midwife delivers a health lecture. Women receive laboratory tests and are encouraged to complete HIV testing at the first visit along with a brief physical assessment with a midwife. The expected number of visits is four.

Group ANC (intervention)

Women enrolled in CP-based group ANC arrive at clinic and go directly to the group space. ANC visits start promptly at the appointment time. The same midwife and co-facilitator are present at each session. Women measure their own vital signs and weight. Each then has a brief one-on-one assessment with the midwife on a mat in a corner of the room. After individual assessments are complete, the midwife and co-facilitator join the circle of women and facilitates interactive discussions using pre-arranged activities. Each session is appropriate for gestational age, but the discussion is fluid~~not rigid~~; women can bring up additional topics and the time allotted can change ~~affect the amount of time per topic~~ by degree of engagement. Each visit

takes place with the same provider and women. Community building occurs over the course of group ANC as women and providers develop trust in each other and explore the common experiences of pregnancy.

Formatted: Highlight

Comment [AM8]: This belongs in the discussion

Formatted: Highlight

The late pregnancy interview and PRES tool was scheduled to coincide with the woman's fourth ANC visit between 32-36 weeks for the individual care group or the fourth group visit for the group care.

Measures

Dependent Variable

The pregnancy-related empowerment scale (PRES) is a 16-item Likert-type scale used to assess women's sense of control over their pregnancy-related health and healthcare. Responses for each item ranged from 1 (strongly disagree) to 4 (strongly agree); the scale has a maximum score of 64. Scale development and content validity, as well as reliability for a sample of pregnant women in the USA, are described by Klima et al [37]. ~~The PRES was collected at the late pregnancy interview that took place between 32-36 weeks gestation.~~ To the best of our knowledge, this is the first study to use the PRES in an African country. The scale had the same internal consistency reliability (α = 0.97) in this context as it had in the U.S.

Formatted: Highlight

Comment [AM9]: This belongs in the discussion

Formatted: Highlight

Independent Variables

Type of care (individual ANC or group ANC), was the primary independent variable for this study. Because both Malawi and Tanzania administer ANC following FANC guidelines, these standards were the same for both countries. Procedures for each type of care were described above.

Based on their well-established association with pregnancy experiences and outcomes, we examined several sociodemographic factors. Age was divided into three groups (<20, 20-34, 35+) since both adolescents and older mothers have a higher risk of complications. ~~Following~~

Comment [AM10]: Reference?

common practice, we also divided women into primigravidas and multiparas. Primigravidas have a somewhat higher obstetric risk and also lack experience with pregnancy, birth and mothering.

Other variables included parity and We included four indicators of socioeconomic status (SES). Education was categorized into three categories (less than primary school, primary school completed, and more than primary school). We also looked at whether the woman said she was a subsistence farmer, indicative of a more rural lifestyle. We also assessed extreme poverty using a single question regarding food insecurity: whether the woman had experienced lack of food or money to buy food in the past four weeks. To obtain some sense of the other end of the economic spectrum in terms of disposable income, we used an assets index, which asks how many of 10 common items a woman's family owns [46]. Religion was included as a sociodemographic factor because it often relates to relationships and reproductive history. We included country as a covariate, a factor because it encapsulates many of the economic and sociodemographic differences between the two countries.

Procedure

Prior to data collection we received necessary approvals from each of three institutional review boards, the University of Illinois at Chicago, College of Medicine Research and Ethics Committee (COMREC) in Malawi, and National Institute for Medical Research (NIMR) in Tanzania. We also received approval from the Ministries of Health and administrators at each participating site. We recruited participants, obtained informed consent and conducted the baseline survey. WomenThen women attended individual ANC or group ANC throughout their pregnancy. Women were contacted between 30-34 weeks gestation to schedule their late pregnancy interview. When possible, repeated telephone contacts were made if a woman did not return for the late pregnancy interview. The baseline and late pregnancy interviews were conducted using the same in-person interview procedures for both group ANC and individual ANC participants. Potential interviewer bias was minimized by extensive training and blinding of interviewers to assignment.

Analysis

We~~To begin, we~~ examined baseline sociodemographic factors of the study participants. We also examined baseline equivalence of the women assigned to group ANC and individual ANC and differences by country, using independent *t* test or χ^2.

General linear modeling (GLM) was employed to assess whether CP-based group ANC led to higher pregnancy-related empowerment (PRES) compared to individual ANC. We adjusted for the sociodemographic covariates plus country and the interaction with country and type of care (Model 1). M~~Then m~~anual backward selection was performed to determine the final reduced model (Model 2) for the full sample.

Because there was a pronounced country by type of care interaction, we then conducted regressions for each country separately, using manual backward elimination on each country's full model to determine the final/reduced model for each country. In Tanzania participants were a mix of Christians and Muslims so we examined the interaction of religion by type of care; this was not done for Malawi because nearly all participants were Christian. We examined the adjusted means by country, religion and type of care using the reduced model analyses conducted by country.

Participants with missing data were excluded through list-wise deletion. However, to examine the impact of differential return rates on results, the GLM results were compared to models estimated using the full information maximum likelihood (FIML) approach to handling missing data. This approach is thought~~known~~ to produce less biased estimates than complete case analyses [47,48]. Using Mplus version 7 [49], we incorporated access to a cell, which was related to missingness, as an auxiliary variable in these inclusive FIML models. We then used imputation to create PRES scores biased to be in the lowest quartile range as well as the highest quartile based on selected subsamples and model covariates except type of care. Imputing missing data to the lowest quartile examined the hypothetical impact on results if all

missing cases had low PRES scores, while imputing to the highest quartile tests the opposite extreme case that all missing cases had high PRES scores.

All GLM analyses, *t-tests*, X^2 tests, and correlations were conducted using version SAS 9.4. Level of significance was set at p<0.05 throughout; because of the small sample size we also discussed trends (p <0.10).

Results

Baseline sociodemographic factors and PRES scores for the entire sample, by type of ANC care and by country are in Table 1.

Table 1. Participant baseline sociodemographic factors and late pregnancy PRES scores

	Full sample (n=192)	Individual ANC (n = 88)	Group ANC (n = 104)		Malawi (n = 91)	Tanzania (n = 101)	
	%	%	%	p-value	%	%	p-value
**Country**							
Malawi	47.4	45.5	49.0	0.62	-	-	-
Tanzania	52.6	54.5	51.0		-	-	-
**Type of Care**							
Control	-	-	-		44.0	47.5	0.62
Group	-	-	-		56.0	52.5	
**Age**							
< 20	13.6	15.1	12.7	0.89	20.2	8.1	**0.04**
20-34	71.0	68.6	70.6		66.3	72.7	
35+	15.4	16.3	16.7		13.5	19.2	
**Gravidity**							
Primigravid	29.6	33.3	26.5	0.30	27.8	31.3	0.60
Multigravid	70.4	66.7	73.5		72.2	68.7	
**Relationship**							
Partner	91.5	96.6	87.3	**0.02**	98.9	84.9	**<0.001**
Single	8.5	3.4	12.7		1.1	15.2	
**Religion**							
Christian	74.6	74.7	74.5	0.97	100.0	51.5	**<0.0001**
Muslim	25.4	25.3	25.5		0.0	48.4	
**Education**							
< Primary	32.2	31.03	33.3	0.74	60.0	7.1	**<0.0001**
= Primary	38.2	43.7	38.2		34.4	46.5	
> Primary	26.98	25.3	28.4		5.6	46.5	
**Occupation**							

Farmer	49.2	49.4	52.0	0.73	97.8	8.1	**<0.0001**
Other	50.8	50.6	48.0		2.2	92.9	
**Food Secure**							
Yes	74.1	75.9	72.5	0.60	66.7	81.1	**0.03**
No	25.9	24.1	27.5		33.3	19.0	
	$\bar{x}$ **(SD)**	$\bar{x}$ **(SD)**	$\bar{x}$ **(SD)**	**p-value**	$\bar{x}$ **(SD)**	$\bar{x}$ **(SD)**	**p-value**
**Assets (0-10)**	3.9 (2.1)	4.1 (2.0)	3.8 (2.1)	0.25	2.6 (1.9)	5.2(1.9)	**<0.0001**
**PRES**	51.5 (8.1)	47.1 (6.4)	55.1 (7.6)	**<0.0001**	52.3 (9.3)	50.7 (6.8)	0.17

With a mean age of 27 (Table 1), most women (71.0%) were between the ages of 20-34. Only 13.6% were less than 20 years and 15.4% were 35 or older. Nearly 30% of the women were primigravidas. Most women (91.5%) were living with their husband or partner. Three-quarters were Christian and one-quarter were Muslim. In terms of education, more than a third of women had not finished primary school (38.2%) completed primary school and just over one-quarter of women (27.0%) had more than a primary school education. Nearly half (49.2%) of the women reported being subsistence farmers, while the others reported other occupations, including housewife, trader, and small business owners. Only a few had higher-level jobs, such as teaching. Just over one-quarter of the women (25.9%) reported experiencing food insecurity in the previous four weeks. Out of 10 common household assets (e.g., bicycle or radio), a mean of 3.9 items was reported by women.

Although more Muslim than Christian women were assigned to group ANC, this was the only significant difference between women in group ANC and individual ANC~~, suggesting that random assignment was effective~~. More women in group ANC than individual ANC were single (12.7% vs, 3.4%, p < 0.022), yet the substantial majority of women in both groups were married or living with a partner.

To provide strong evidence about the robustness of the group ANC model in different sectors of Eastern and Southern Africa, we chose to two challenging settings in sub-Saharan countries. Although Malawi and Tanzania are both low-resource countries with many broad similarities in their healthcare systems and ANC protocols, overall, Malawi has less favorable

Table 2. PRES item means (SD), total scores for the sample and by type of care*

economic indicators. Therefore, we also examined country differences for all independent variables (Table 1). There were more than twice as many adolescent mothers in the Malawi sample (22.2% vs. 8.1%). Differences also existed in whether women were currently in a relationship. With the exception of one woman, all in Malawi were Christian. In Tanzania about half were Muslim. Women in Malawi were more likely to report being farmers (98% versus 8% p=0.0001). They also reported more food insecurity (33% in Malawi and 19% in Tanzania, p = 0.03) and had roughly half the number of assets than women in Tanzania. Last, there was a major difference between the two countries in the way that the CP-based group ANC was implemented. In Malawi the sessions lasted nearly twice as long as they did in Tanzania, an average of 4.0 (SD 0.4) hours compared to only 2.3 (SD 0.2) hours in Tanzania.

Next we examined overall PRES scores and individual PRES scale items for the whole sample and by type of care. The mean PRES score for the entire sample was 51.5 (SD 8.1). The mean PRES score was significantly higher for women in group ANC compared to those in individual ANC (55.1 v. 47.1, respectively) and women in group ANC also had significantly higher scores on every item (Table 2). Another interesting observation was that range in scores was quite different by country and type of care. Many women in group ANC in Malawi had the highest possible score (n = 18, 69%). We also explored variation by country and type of care in distribution of PRES score. 22 women (64.7%) in group ANC in Malawi had the maximum PRES score of 64, whereas none of the Malawian women in individual ANC had the maximum score. In Tanzania, the maximum score was reported by just 9 women (26.5%) in group ANC and only 3 women (8.8%) in individual ANC.

	Full sample	Individual ANC	Group ANC
**Provider Connectedness**	n=192	n=88	n=104
I can ask my midwife provider about my pregnancy.	3.31 (0.57)	3.02 (0.48)	3.55 (0.52)
I have enough time with my midwife to discuss my pregnancy.	3.18 (0.64)	2.83 (0.60)	3.48 (0.52)
My midwife listens to me.	3.23 (0.64)	2.87 (0.60)	3.52 (0.50)
My midwife respects me.	3.25 (0.58)	2.94 (0.51)	3.51 (0.50)
I expect my midwife to respect my decisions about my pregnancy.	3.23 (0.58)	2.93 (0.50)	3.47 (0.52)
My midwife respects my decision, even if it is different than her/his recommendation.	3.03 (0.74)	2.80 (0.63)	3.22 (0.78)
**Skillful Decision-Making**			
I take responsibility for the decisions I make about my pregnancy like eating healthy food.	2.95 (0.89)	2.69 (0.75)	3.17 (0.94)
I can tell when I have made a good health choice.	3.27 (0.53)	3.05 (0.48)	3.46 (0.50)
Since I began prenatal care, I have been making more decisions about my health.	3.27 (0.53)	3.05 (0.48)	3.46 (0.50)
**Peer Connectedness**			
Women need to share experiences with other women when they are pregnant.	3.28 (0.60)	3.05 (0.61)	3.48 (0.50)
I share my feelings and experiences with other women.	3.21 (0.60)	2.94 (0.60)	3.43 (0.50)
**Gaining Voice**			
I know if I am gaining the right amount of weight during my pregnancy.	3.16 (0.67)	2.81 (0.66)	3.46 (0.50)
I have a right to ask questions when I don't understand something about my pregnancy.	3.23 (0.66)	2.92 (0.68)	3.50 (0.50)
I am able to change things in my life that are not healthy for me.	3.25 (0.57)	3.00 (0.53)	3.46 (0.52)
I am doing what I can to have a healthy baby.	3.31 (0.55)	3.11 (0.54)	3.48 (0.50)
If something is going wrong in my pregnancy, I know who to talk to.	3.32 (0.53)	3.13 (0.50)	3.48 (0.50)
**Total PRES Score**	**51.46 (8.10)**	**47.12 (6.43)**	**55.13 (7.56)**

***All items comparing individual ANC and group ANC were significantly different, p <.0001**

We then examined the impact of type of care on PRES using regression for the total sample. In the crude model without controlling for other factors, type of care alone explained 24% of the variation in PRES scores. In Model 1, we included all of the factors reported in Table 1 and an interaction term for country and type of care. Earlier bivariate analyses had identified that the demographics for women were very different by country. In particular, there was only one Muslim woman in the Malawi sample. In the full model with the interaction term, the explained variance increased to 46.54% (**Table 3, Model 1**). Type of care remained a significant predictor of PRES along with country, religion, and the interaction term.

Comment [AM11]: I feel like this needs to be explained more or deleted, most readers are not familiar with this.

Using backward elimination, we then identified a final regression model predicting PRES (Full sample, Model 2). Predictors in the final model were type of care ($p < 0.0001$), country ($p < 0.0001$) country by type of care ($p < 0.0001$), and religion ($p < 0.05$), which together explained 44.87% of the variation in PRES scores.

Table 3. Predictors of PRES for the full sample and by country

			By Country[a]			
	Full Sample[a]		Malawi		Tanzania	
	Model 1	Model 2	Model 1	Model 2	Model 1	Model 2
**Variable**	*B*	*B*	*B*	*B*	*B*	*B*
Intercept	42.09 (3.25)***	43.64 (0.97)***	48.84 (7.39)***	43.70 (0.84)***	49.17 (3.54)***	50.20 (1.32)***
Type of Care (Group ANC)	15.19 (1.34)***	15.31 (1.30)***	15.17(1.24)***	15.26 (1.13)***	-0.24 (2.05)	-0.47 (1.85)
Country (Tanzania)	8.36 (2.50)**	7.40 (1.43)***	-	-	-	-
Type of Care/ Country interaction	-13.29 (1.88)***	-13.52 (1.78)***	-	-	-	-
Religion (Muslim)	-2.33 (1.31)*	-2.68 (1.22)**	-	-	-5.03 (2.21)**	-5.15 (1.93)**
Type of Care/ Religion interaction[b]	-	-	-	-	4.76 (3.01)	4.69 (2.66)*
Education < primary)	-1.07 (1.63)		-1.73 (2.81)	-	-1.68 (3.06)	-
Education (≥ primary)	-0.07 (1.27)		-1.03 (2.78)	-	-0.10 (1.60)	-
Age <20	1.09 (1.62)		0.38 (2.12)	-	0.77 (2.96)	-
Age 35+	0.95 (1.31)		2.15 (1.85)	-	-0.18 (1.99)	-
Gravidity (Multiparous)	1.25 (1.25)		-0.06 (1.91)	-	1.87 (1.78)	-
Partner (Yes)	0.28 (1.83)		-4.02 (5.83)	-	0.98 (2.23)	-
Farmer/Herder	1.44 (2.13)		1.50 (4.24)		1.69 (2.87)	-
Food Secure (Yes)	0.57 (1.08)		1.46(1.27)	-	-0.67 (1.97)	-
Assets	-0.18 (0.30)		-0.41 (0.49)	-	0.11 (0.42)	-
R^2	0.4654	0.4487	.6925	0.6776	.1051	.0851

* p <.10, **p<.05, ***p<.0001
[a] Standard errors displayed in parentheses
[b] The interaction for type of care and religion was only included in Tanzania models because there was no variation in religion in Malawi

To further clarify the joint impacts of type of care, country and religion, we conducted a stratified analysis to examine type of care and the predictors of empowerment separately for

each country (**Table 3**). For Malawi, the full model with the predictive covariates (Model 1) explained 69.25% of the variation (note there was no variability in religion in Malawi so we did not adjust for it). In Malawi, only type of care was a significant predictor. Not surprisingly, backward elimination produced a final model for Malawi that was the same as the crude model, with only type of care as a predictor. Type of care explained 67.76% of the variation in PRES in the final model (Model 2).

The picture was different for urban pregnant women in Tanzania, half of whom were Muslim. The full model included type of care, all predictive factors and the interaction of religion and type of care. The full model only explained 10.5% of the variation in empowerment. Religion ($p < 0.05$) was the only significant predictor. Type of care and religion interaction was only marginally significant ($p < 0.1$). Type of care was non-significant. The final model included type of care, religion, and religion by type of care, which explained 8.51% of the variation in PRES scores.

We compared the adjusted mean PRES scores for women in individual ANC to those in group ANC by country and, in Tanzania, by religion (see Model 2 for Malawi and Model 2 for Tanzania in Table 3). In Malawi, the adjusted mean PRES score was 59.06 and 43.7 for women in group ANC and individual ANC, respectively ($p < 0.001$). In Tanzania, the adjusted means for type of care by religion show an interesting pattern (**Figure 2**). For Christians, there was no difference in PRES scores by type of care ($p = 0.799$). However among Muslims, women in CP-based group ANC had a mean PRES score of 51.27, while those in individual ANC had a score of 47.05. This difference was statistically significant for Muslims in the reduced model ($p = .02$), suggesting that group ANC affected pregnancy-related empowerment differently for Muslim women than for Christian women in urban Tanzania.

-- INSERT FIGURE 2 APPROXIMATELY HERE --

To further explore the differences between Muslim and Christian women in Tanzania, we looked at the sociodemographic differences to assess how these might relate to the difference

Formatted: Highlight

Comment [AM12]: This paragraph needs to be rewritten, it is very confusing. Simplify it – I think it says that for urban women in Tanzania, there was no difference in empowerment scores regardless of type of care.

Formatted: Highlight

in the effect that type of care had on PRES scores. Muslim women were more likely to be food insecure (26.53% vs 11.76%, p = 0.0599) and more likely to be among the youngest age group <20 years (16% vs 0%, p = 0.0063).

To examine the effects of differential loss to follow-up, GLM results were compared to models estimated using the FIML approach to handling missing data. These FIML models, using all participants and available data, produced similar parameter estimates and consistent statistical conclusions across all models presented.

Using imputation of missing data, we then examined the hypothetical impact on results if all missing cases had either extremely high or extremely low PRES scores. In these fully adjusted models, our findings were robust for the effect of type of care (p<.0001 in both models). For the model with missing imputed as a low PRES score, the adjusted means for type of care were 43.3 for individual ANC and 57.7 for group ANC. For the model with missing imputed as a high PRES score, the adjusted means for group ANC were 59.4 and 49.9 for individual ANC.

Discussion

The expectation that women in CP-based group ANC would have higher pregnancy-related empowerment scores in late pregnancy was only partially confirmed in this study. Group ANC was strongly related to higher pregnancy-related empowerment in Malawi, but not in Tanzania. In Malawi, type of care was the only predictor of PRES scores. In Tanzania, Muslim women in group ANC had significantly higher adjusted PRES scores than Muslim women in individual ANC; however, type of care did not relate to pregnancy-related empowerment among Christian women.

There are several possible factors that might have contributed to the observed pattern of differences in pregnancy-related empowerment. These explanations are somewhat speculative given our small sample and the lack of a rural sample in Tanzania or an urban sample in Malawi. One reason that type of care may have affected pregnancy-related empowerment in

Malawi, but not in Tanzania, that urban women have a wider range of opportunities [50]. However, if the major reason for the pattern of finding was urban-rural differences, we would expect all of the women in the urban sample would have experienced the same effect from group ANC. Instead, only Muslim women in Tanzania had higher pregnancy-related empowerment in group care. This may be related to the sociodemographic factors putting Muslim women in this sample at a disadvantage, including younger age and more food insecurity. Our findings are congruent with U.S. studies showing that CP benefitted more disadvantaged women [51,52]. Lower levels of autonomy in health-related decision making among Muslims have been reported in other African countries [53–55].

In addition to urban-rural differences, another potential explanation may be related to the length of group sessions. Women in group ANC in Malawi received nearly twice as much contact time per session than women in Tanzania, where sessions lasted approximately two hours. In Malawi, the CP-based group ANC model was implemented with flexibility, and sessions continued until all issues were discussed. Although this made the sessions longer than intended, the more time devoted to interactive learning in Malawi may have contributed to greater pregnancy-related empowerment. In the context of four recommended antenatal visits in both countries, ANC clients might benefit from the additional discussion time, either as longer session or an increase in the number of ANC visits, especially since the number of ANC visits is considerably higher in most high-income countries [56,57]. The issue of the optimal number of ANC visits and contact time during pregnancy certainly requires a second look [58].

A third possible factor is that group ANC may not have as profound an effect in clinics where the care is already perceived as high quality. The site in Dar es Salaam has a reputation for being one of the better government facilities in the city. Since women make decisions about health services based on perceived quality [59], the women who chose to come to this clinic may have already had higher pregnancy-related empowerment.

Limitations

A major limitation of this pilot was the lack of comparable urban and rural sites in both countries. We had initially planned to have four sites, one rural and one urban in each country; however, ~~-~~ ~~However,~~ funding constraints forced us ~~meant we had~~ to limit the study. We felt it was critically important to examine whether group ANC could be implemented successfully in both rural and urban settings. Large urban metropolises, such as Dar es Salaam in Tanzania, offer many unique challenges. We also wanted to examine whether group ANC could work in settings with severely limited resources for both the health system and pregnant women. The substantially greater national poverty in Malawi made it an ideal setting~~-~~for study. ~~to assess this~~. This design provided strong evidence regarding the robustness of the CP-based ANC model in two very different settings. However, because of this design choice, we are unable to disentangle the urban-rural and country differences. A larger RCT will allow for ~~a fuller~~ exploration of these issues.

A second limitation is that we only collected PRES data once in pregnancy. Because the PRES focused on ANC experiences, it was not appropriate to ask these questions at baseline. However, measurement of pregnancy-related empowerment at multiple time points in pregnancy would allow examination of whether PRES changes over the course of pregnancy and whether these changes are related to type of care.

Last, differences between groups due to attrition bias are another potential limitation. However, the two approaches we used to examine the impact of missing data suggested missing data had minimal effect on the results of this study.

Implications

This study provides evidence that ANC models may affect pregnancy-related empowerment in some contexts ~~but not in others~~. These pilot results indicate that in a rural setting in Malawi where poverty is high, a CP-based group ANC model was associated with higher levels of pregnancy empowerment. However, in an urban setting in Tanzania, the same group ANC model only related to higher empowerment among Muslim women.

Education, socioeconomic status, parity, and partner status minimally related to pregnancy-related empowerment in this study. These results suggest that pregnancy-related empowerment is a distinct psychosocial phenomenon that does not just reflect sociodemographic factors.

Pregnancy-related empowerment is important in sub-Saharan Africa, where low quality ANC and severe health worker shortages contribute to poor maternal and infant outcomes. CP-based group ANC is a promising model to address these challenges and to increase pregnancy-related empowerment for women in some contexts. More research is needed to further assess the relationship of empowerment, type of ANC, and health-related outcomes for pregnant women globally.

Comment [AM13]: Reference?

Acknowledgements:

Funding for this pilot was provided by The National Institute for Nursing Research, National Institutes of Health (Grant NR014413). The hard work, dedication and support of our Tanzanian (Lugano Mafwenga, Dorkas Mwakawanga, Peter Shirima, Willy Sangu) and Malawi Teams (Mercy Katukumala, Chimwemwe Chamthinya, and Ekari Chitema) made this pilot run smoothly. We gratefully acknowledge the many hours that Jeslyn Koovakada spent readying the data for analyes. Feedback from members of the ministries of health, administrators, midwives, health workers and the women was critical throughout the process; their input is gratefully acknowledged.

Reviewer 2: Joelle Brown

In general, I thought the revised manuscript was improved, and think that the authors adequately addressed many of the questions raised by the reviewers.

However, I still think (for the reasons I stated in my original set of comments) that presenting the overall results is potentially misleading and that presenting the results by country is more appropriate. I did not follow the author's argument that there is merit in presenting the combined analysis. My concern is that presenting the combined analysis as the primary result hides the important differences seen in the effect of the intervention across sites: the intervention appeared to have an effect in Malawi but not in Tanzania. That difference in effect is an important and interesting finding. Had there not been such significant differences in the effect of the intervention across country, then presenting the combined analysis may have had more value.

Follow-up comments based on the current draft:

Location in paper	Major Comments
Abstract/Main Results	I think it is potentially misleading to present the overall effect of the PRES intervention as the main finding when it is clear from the data presented that the effects of the intervention differed significantly in the individual countries. I still think the overall analysis should be dropped, or if included, minimized. Not only did the intervention have significantly different effects in the two countries, there were other major difference in the two sites that call into question the appropriateness of collapsing the data, including that the interventions were delivered differently in the two settings, one site was urban vs rural, and the participants in the two countries differed significantly by age, religion, relationship status, and SES.
Methods, line 133	For an RCT, you will need to provide more detail on the randomization procedure, especially how the randomization list was generated and how the allocation was concealed until the time of randomization.<br><br>The reason for needing this information is that the validity and credibility of the study findings are highly linked to how successful the randomization process was. Readers need to know how rigorous the randomization technique was and whether study staff could have in any way subverted the randomization and allocated women to group or individual ANC. Or whether women could have self-selected to be in group vs individual ANC.<br><br>For example, please clarify who created the randomization list? Was a computer generated sequence used? How were envelopes selected? Were envelopes numbered and assigned sequentially as women came into the clinic, or did women select an envelope out of a hat? Or did staff select an envelope for individuals? Was there any switching of group assignment?<br><br>If randomization was 1:1 (CP group ANC:individual ANC), then state that.
Methods, lines 141-146	These results belong in the results, not the methods.
Methods, Line 148	It is not clear what is meant in the second half of the following sentence: "For women in group ANC, the research team knew when their last group visit was scheduled, so the team arranged to interview them even if we could not reach them by cell phone." Please clarify in the paper
Methods, line 151	How long were the individual ANC sessions meant to be?
Methods, line 161	In general, more information is needed on the intervention itself: How long were the brief

	one-on-one sessions with the midwife meant to be? How long were the group sessions meant to be?
Methods, line 208-211	Please clarify who did the interviewing? For example, was it the clinic nurses who provided ANC, or study staff separate from the ANC provision?<br><br>In what language(s) were the interviews conducted?<br><br>Were the women randomized to group ANC interviewed as individuals? Again, by whom were they interviewed? And in what language?
Methods, line 216	More detail on the statistical methods would be useful.
Methods, line 216	Did your analysis need to account for clustering by group ANC? If not, why not?
Results, lines 257-261	This paragraph in the results disagrees with the data in Table 1.<br><br>In table one 'single vs. partner' is the only characteristic that is different between individual and group care. There is no difference in religion by randomization group<br><br>Also make sure the data in the results paragraphs are the same as the data you present in the tables. Please correct.
Results, lines 264	Please also include the average length of time (SD) for the individual sessions.
Results, line 281-284	These two sentences seem contradictory and duplicative: "Many women in group ANC in Malawi had the highest possible score (n = 18, 69%). 22 women (64.7%) in group ANC in Malawi had the maximum PRES score of 64"
Table 1	Why did you decide to summarize baseline sociodemographics for only those who were retained (n=192) rather than the total sample that was randomized (n=218)?<br><br>Typically, Table 1 describes all randomized participants, not just those that are retained at the end of the study.
Table 3	Please explain in the title or header of Table 3 what the values in Table 3 refer to.<br><br>Please include the 'n' in each column.
Figure 2	Figure 2 should be corrected so that the maximum number is 64, not 70, assuming that the PRES scale has a maximum of 64. Please include the confidence intervals in Figure 2.
	Minor comments
Abstract, line 31	I would recommend defining CP the first time it appears in the abstract, or taking the acronym out of the background of the abstract.

Reviewer 3: Donatello Telesca

Statistical Analysis Feedback

I the following I will comment on some of the referee's points that pertain the statistical analysis. I am also adding some general comments for your consideration.

Below I will highlight my feedback in bold.

Location in paper	Major Comments
Abstract/Main Results	I think it is potentially misleading to present the overall effect of the PRES intervention as the main finding when it is clear from the data presented that the effects of the intervention differed significantly in the individual countries. I still think the overall analysis should be dropped, or if included, minimized. Not only did the intervention have significantly different effects in the two countries, there were other major difference in the two sites that call into question the appropriateness of collapsing the data, including that the interventions were delivered differently in the two settings, one site was urban vs rural, and the participants in the two countries differed significantly by age, religion, relationship status, and SES.<br><br>**DT: I agree with the referee. It seems as thought the two populations, in Malawi and Tanzania, have significantly different characteristics. Because you are working with a RCT, I would simply report as the main result, two separate analyses for the two countries, where type of care is the only predictor. Alternatively, if similar error variances are expected in the two countries, you may consider a model with country, type of care and interactions as predictors. Subsequent analyses involving adjustment by other explanatory factors could be considered secondary to the main scope of the manuscript.**
Methods, line 133	For an RCT, you will need to provide more detail on the randomization procedure, especially how the randomization list was generated and how the allocation was concealed until the time of randomization.<br><br>The reason for needing this information is that the validity and credibility of the study findings are highly linked to how successful the randomization process was. Readers need to know how rigorous the randomization technique was and whether study staff could have in any way subverted the randomization and allocated women to group or individual ANC. Or whether women could have self-selected to be in group vs individual ANC.<br><br>For example, please clarify who created the randomization list? Was a computer generated sequence used? How were envelopes selected? Were envelopes numbered and assigned sequentially as women came into the clinic, or did women select an envelope out of a hat? Or did staff select an envelope for individuals? Was there any switching of group assignment?<br><br>If randomization was 1:1 (CP group ANC:individual ANC), then state that.
Methods, lines 141-146	These results belong in the results, not the methods.
Methods, Line 148	It is not clear what is meant in the second half of the following sentence: "For women in group ANC, the research team knew when their last group visit was scheduled, so the team arranged to interview them even if we could not reach them by cell phone." Please clarify in the paper
Methods, line 151	How long were the individual ANC sessions meant to be?
Methods, line 161	In general, more information is needed on the intervention itself: How long were the brief one-on-one sessions with the midwife meant to be? How long were the group sessions meant to be?
Methods, line 208-211	Please clarify who did the interviewing? For example, was it the clinic nurses who provided ANC, or study staff separate from the ANC provision?<br><br>In what language(s) were the interviews conducted?<br><br>Were the women randomized to group ANC interviewed as individuals? Again, by whom were they interviewed? And in what language?

Methods, line 216	More detail on the statistical methods would be useful.<br><br>**DT: There is indeed no description of statistical methods used. In the manuscript, the analysis method is simply identified as GLM. The term GLM is used to refer to an entire family of probability distributions. To make the context of analytical results more clear you should indicate precisely what kind of GLM was used to draw scientific conclusions and why. Furthermore, in the backward selection strategy you should indicate what scoring method was used to select the final model/s.**
Methods, line 216	Did your analysis need to account for clustering by group ANC? If not, why not?<br><br>**DT: I think clustering should be accounted for in your analysis. In the administration of group care, it is conceivable that women sharing the same provider may experience similar results. An analysis accounting for this kind of grouping would indeed be required for valid statistical inference. Mixed effects models or GEE methods would be the way to proceed.**
Results, lines 257-261	This paragraph in the results disagrees with the data in Table 1.<br><br>In table one 'single vs. partner' is the only characteristic that is different between individual and group care. There is no difference in religion by randomization group<br><br>Also make sure the data in the results paragraphs are the same as the data you present in the tables. Please correct.
Results, lines 264	Please also include the average length of time (SD) for the individual sessions.
Results, line 281-284	These two sentences seem contradictory and duplicative: "Many women in group ANC in Malawi had the highest possible score (n = 18, 69%). 22 women (64.7%) in group ANC in Malawi had the maximum PRES score of 64"
Table 1	Why did you decide to summarize baseline sociodemographics for only those who were retained (n=192) rather than the total sample that was randomized (n=218)?<br><br>Typically, Table 1 describes all randomized participants, not just those that are retained at the end of the study.<br><br>**DT: I agree with the referee. Ideally Table 1 should serve as a sanity check for the results of randomization, therefore all study participants should contribute to the summaries.**
Table 3	Please explain in the title or header of Table 3 what the values in Table 3 refer to.<br><br>Please include the 'n' in each column.
Figure 2	Figure 2 should be corrected so that the maximum number is 64, not 70, assuming that the PRES scale has a maximum of 64. Please include the confidence intervals in Figure 2.
	Minor comments
Abstract, line 31	I would recommend defining CP the first time it appears in the abstract, or taking the acronym out of the background of the abstract.

General Considerations

1) Because you are working with a RCT, I don't see the need to adjust for other baseline predictors in your main analysis. Dividing the analysis by country makes sense, as you are dealing with two different target populations.
2) Because type of care in the experimental group is administered in a group setting, some care is needed in the formulation of statistical models that assume independently observed outcomes. Your data is complicated by the fact that, clustering is present in the treatment group, but not in the control group. This fact may have to be addressed with more sophisticated modeling strategies involving random effects.
3) Backward variable selection is statistically deprecated. I would encourage you to consider all subset selection, if you pursue a predictive analysis aimed at including many predictors.

Response to reviewers
– 2nd round

First Reviewer
Reviewer: Meg Autry
We accepted Meg Autry's editorial suggestions and comments she made using track changes.

Second Reviewer
Reviewer: Joelle Brown
Comments on revised PRES manuscript draft
In general, I thought the revised manuscript was improved, and think that the authors adequately addressed many of the questions raised by the reviewers. However, I still think (for the reasons I stated in my original set of comments) that presenting the overall results is potentially misleading and that presenting the results by country is more appropriate. I did not follow the author's argument that there is merit in presenting the combined analysis. My concern is that presenting the combined analysis as the primary result hides the important differences seen in the effect of the intervention across sites: the intervention appeared to have an effect in Malawi but not in Tanzania. That difference in effect is an important and interesting finding. Had there not been such significant differences in the effect of the intervention across country, then presenting the combined analysis may have had more value.
We now present the results by country only.

Statistical Analysis Feedback
Donatello Telesca (UCLA Biostatistics)
I the following I will comment on some of the referee's points that pertain to the statistical analysis. I am also adding some general comments for your consideration. Below I will highlight my feedback in bold.
The combined table below shows that we addressed both Joelle and Donatello's comments. Our responses are in blue below DT's comments.

Location in paper	Major Comments
Abstract/Main Results	I think it is potentially misleading to present the overall effect of the PRES intervention as the main finding when it is clear from the data presented that the effects of the intervention differed significantly in the individual countries. I still think the overall analysis should be dropped, or if included, minimized. Not only did the intervention have significantly different effects in the two countries, there were other major difference in the two sites that call into question the appropriateness of collapsing the data, including that the interventions were delivered differently in the two settings, one site was urban vs rural, and the participants in the two countries differed significantly by age, religion, relationship status, and SES.<br>**DT: I agree with the referee. It seems as though the two populations, in Malawi and Tanzania, have significantly different characteristics. Because you are working with a RCT, I would simply report, as the main result, two separate analyses for the two countries, where type of care is the only predictor. Alternatively, if similar error variances are expected in the two**

	**countries, you may consider a model with country, type of care and interactions as predictors. Subsequent analyses involving adjustment by other explanatory factors could be considered secondary to the main scope of the manuscript.**<br><br>**After completing a full analysis, we found that the PRES was the only variable with these country differences. We dropped the overall analysis from this paper and present the country results.**
Methods, line 133	For an RCT, you will need to provide more detail on the randomization procedure, especially how the randomization list was generated and how the allocation was concealed until the time of randomization.<br><br>The reason for needing this information is that the validity and credibility of the study findings are highly linked to how successful the randomization process was. Readers need to know how rigorous the randomization technique was and whether study staff could have in any way subverted the randomization and allocated women to group or individual ANC. Or whether women could have self-selected to be in group vs individual ANC.<br><br>For example, please clarify who created the randomization list? Was a computer generated sequence used? How were envelopes selected? Were envelopes numbered and assigned sequentially as women came into the clinic, or did women select an envelope out of a hat? Or did staff select an envelope for individuals? Was there any switching of group assignment?<br>**We describe this process in more detail and state that randomization was concealed.**<br><br>If randomization was 1:1 (CP group ANC:individual ANC), then state that.<br>**Randomization was 1:1. Clarified**
Methods, lines 141-146	These results belong in the results, not the methods.<br>**Moved as suggested**
Methods, Line 148	It is not clear what is meant in the second half of the following sentence: "For women in group ANC, the research team knew when their last group visit was scheduled, so the team arranged to interview them even if we could not reach them by cell phone." Please clarify in the paper<br>**Section rewritten**
Methods, line 151	How long were the individual ANC sessions meant to be?<br>**Original line 151 referred to group ANC; we added more detail about group care sessions.**
Methods, line 161	In general, more information is needed on the intervention itself: How long were the brief one-on-one sessions with the midwife meant to be? How long were the group sessions meant to be?<br>**Added clarifying detail about the intervention. We also cited a**

	**newly published book that describes the model fully (Rising and Quimby, 2016).**
Methods, line 208-211	Please clarify who did the interviewing? For example, was it the clinic nurses who provided ANC, or study staff separate from the ANC provision?<br>**Clarified**<br>In what language(s) were the interviews conducted?<br>**Added (Chichewa in Malawi; Swahili in Tanzania)**<br>Were the women randomized to group ANC interviewed as individuals? Again, by whom were they interviewed? And in what language?<br>**Clarified**
Methods, line 216	More detail on the statistical methods would be useful.<br>**DT: There is indeed no description of statistical methods used. In the manuscript, the analysis method is simply identified as GLM. The term GLM is used to refer to an entire family of probability distributions. To make the context of analytical results more clear you should indicate precisely what kind of GLM was used to draw scientific conclusions and why. Furthermore, in the backward selection strategy you should indicate what scoring method was used to select the final model/s.**<br>**Description was expanded and reflects the analytical approach.**
Methods, line 216	Did your analysis need to account for clustering by group ANC? If not, why not?<br>**DT: I think clustering should be accounted for in your analysis. In the administration of group care, it is conceivable that women sharing the same provider may experience similar results. An analysis accounting for this kind of grouping would indeed be required for valid statistical inference. Mixed effects models or GEE methods would be the way to proceed.**<br>**While the intervention was delivered in groups for half the participants, within each setting the study was conducted as a randomized controlled trial with randomization at the individual level. By design, there should be no clustering effects that need to be controlled for baseline characteristics. At each site, midwives who provided group care may have also provided individual care and we do not have a record of that. Also, there is rarely continuity of care for those in individual ANC, so clustering effects by provider would be minimal for individual ANC. We ran the models accounting for provider effects for group participants using GEE models with individual ANC participants coded as clusters of 1. We found results that are consistent with those in the manuscript.**
Results, lines 257-261	This paragraph in the results disagrees with the data in Table 1.<br>**Corrected**<br>In table one 'single vs. partner' is the only characteristic that is different between individual and group care. There is no difference in religion by randomization group

	**Corrected**<br>Also make sure the data in the results paragraphs are the same as the data you present in the tables. Please correct.<br>**Corrected**
Results, lines 264	Please also include the average length of time (SD) for the individual sessions.<br>**Clarified**
Results, line 281-284	These two sentences seem contradictory and duplicative: “Many women in group ANC in Malawi had the highest possible score (n = 18, 69%). 22 women (64.7%) in group ANC in Malawi had the maximum PRES score of 64”<br>**Corrected**
Table 1	Why did you decide to summarize baseline sociodemographics for only those who were retained (n=192) rather than the total sample that was randomized (n=218)?<br>**Corrected**<br>Typically, Table 1 describes all randomized participants, not just those that are retained at the end of the study.<br>**Corrected**<br>**DT: I agree with the referee. Ideally Table 1 should serve as a sanity check for the results of randomization, therefore all study participants should contribute to the summaries.**<br>**Corrected**
Table 3	Please explain in the title or header of Table 3 what the values in Table 3 refer to.<br>**See new tables**<br>Please include the ‘n’ in each column.<br>**These data are Table 3 (Mean (SD) PRES scores by country). Table 4 shows the predictive models of PRES for each country.**
Figure 2	Figure 2 should be corrected so that the maximum number is 64, not 70, assuming that the PRES scale has a maximum of 64. Please include the confidence intervals in Figure 2.<br>**Figure 2 was removed**
	Minor comments
Abstract, line 31	I would recommend defining CP the first time it appears in the abstract, or taking the acronym out of the background of the abstract.<br>**Done**

General Considerations

- **Because you are working with a RCT, I don’t see the need to adjust for other baseline predictors in your main analysis. Dividing the analysis by country makes sense, as you are dealing with two different target populations.**

We reported t-tests for treatment arm differences with no adjustment for covariates.

- **Because type of care in the experimental group is administered in a group setting, some care is needed in the formulation of statistical models that assume independently observed outcomes. Your data is complicated by the fact that, clustering is present in the treatment group, but not in the control group. This fact may have to be addressed with more sophisticated modeling strategies involving random effects.**

Since randomization is at the individual level, there should be no clustering effects.

- **Backward variable selection is statistically deprecated. I would encourage you to consider all subset selection, if you pursue a predictive analysis aimed at including many predictors.**

We have included subset selection models secondary to the main effect results.
